# A GDF-15–GFRAL axis controls autoimmune T cell responses during neuroinflammation

**DOI:** 10.1038/s41590-025-02406-1

**Published:** 2026-01-15

**Authors:** Jana K. Sonner, Audrey Kahn, Lars Binkle-Ladisch, Jan Broder Engler, Beatrice Haack, Christina Zeiler, Lisa Unger, Simone Bauer, Felix Fischbach, Giovanni Almanzar, Mark Walkenhorst, Christina Mayer, Aneta Kolakowska, Sebastian Graute, Caren Ramien, Ingo Winschel, Nicola Rothammer, Markus Heine, Verena Horneffer-van der Sluis, Vincent Thiemann, Vanessa Vieira, Nina Meurs, Thomas Renné, Martina Prelog, Sebastian Beck Jørgensen, Randy J. Seeley, Anke Diemert, Petra C. Arck, Stefan M. Gold, Joerg Heeren, Jörg Wischhusen, Manuel A. Friese

**Affiliations:** 1 Institute of Neuroimmunology and Multiple Sclerosis, University Medical Center Hamburg-Eppendorf, Hamburg, Germany; 2 Hamburg Center for Translational Immunology, University Medical Center Hamburg-Eppendorf, Hamburg, Germany; 3 Experimental Tumor Immunology, Department of Obstetrics and Gynecology, University Hospital Würzburg, Würzburg, Germany; 4 Department of Pediatrics, Pediatric Rheumatology/Special Immunology, University Hospital Würzburg, Würzburg, Germany; 5 Department of Biochemistry and Molecular Cell Biology, University Medical Center Hamburg-Eppendorf, Hamburg, Germany; 6 Institute of Clinical Chemistry and Laboratory Medicine, University Medical Center Hamburg-Eppendorf, Hamburg, Germany; 7 Center for Thrombosis and Hemostasis (CTH), Johannes Gutenberg University Medical Center, Mainz, Germany; 8 Irish Centre for Vascular Biology, School of Pharmacy and Biomolecular Sciences, Royal College of Surgeons in Ireland, Dublin, Ireland; 9 Bio Innovation Hub Transformational Research Unit, Novo Nordisk, Boston, MA USA; 10 Department of Surgery, University of Michigan, Ann Arbor, MI USA; 11 Department of Obstetrics and Fetal Medicine, University Medical Center Hamburg-Eppendorf, Hamburg, Germany; 12 Department of Psychiatry and Medical Department, Campus Benjamin Franklin, Charité-Universitätsmedizin Berlin, Berlin, Germany

**Keywords:** Neuroimmunology, Multiple sclerosis, Translational immunology, T cells

## Abstract

Inflammatory activity during multiple sclerosis (MS) often improves during pregnancy, suggesting that pregnancy-related immune adaptations affect the disease. Here we show that growth/differentiation factor-15 (GDF-15) increases during pregnancy and correlates with a reduced rate of MS relapses. GDF-15 also accumulates in the inflamed central nervous system, and its absence impairs inflammation resolution in a mouse model of MS. GDF-15 suppresses autoimmune T cell responses through an indirect signaling pathway involving the activation of GDNF family receptor α-like (GFRAL) on brainstem neurons. Therapeutic approaches, including neuronal gene delivery, recombinant GDF-15 administration and targeted chemogenetic activation of GFRAL-positive neurons induce β-adrenergic signaling and norepinephrine synthesis in the spleen, leading to decreased expression of integrins on T cells required for transmigration across the blood–brain barrier and confer protection against neuroinflammation in preclinical models of MS. These findings position GDF-15 as a crucial neuroimmune mediator and the GDF-15–GFRAL axis as promising target for MS.

## Abstract (web summary)

Here the authors show that endogenous or therapeutically delivered GDF-15 activates brainstem neurons that trigger splenic β-adrenergic signaling. This, in turn, suppresses autoreactive T cells and reduces neuroinflammation, identifying a possible target for multiple sclerosis treatment.

## Main

MS is a common neuroinflammatory disorder that primarily affects individuals in early adulthood. It likely arises from a dysregulated immune response directed against central nervous system (CNS) self-antigens, leading to inflammatory CNS lesions. Autoreactive T cells cross the blood–brain barrier, activate CNS-resident myeloid cells and drive progressive demyelination and neurodegeneration^1,2^. To counteract tissue inflammation, the body relies on inherent mechanisms of immune tolerance.

During pregnancy, a substantial reduction in inflammatory disease activity is observed in MS^3,4^ and other T cell-mediated autoimmune diseases, such as rheumatoid arthritis or Graves’ disease^5^, emphasizing the potent immunomodulatory impact of pregnancy—effects partly reproduced in animal models^6–8^. Treatment with the pregnancy hormone estriol is effective in autoimmune encephalomyelitis^9^ and shows some effects in clinical trials of nonpregnant women with MS, but does not fully recapitulate the pregnancy-related reduction in relapse rates^10^. This suggests that a more complex regulatory network governs transient tolerance during pregnancy. Elucidating the key mechanisms behind this evolutionarily selected immune tolerance, which allows the maternal body to accept the semi-allogeneic fetus, could be crucial for developing new therapeutic strategies for immune-mediated inflammatory diseases.

Bidirectional communication between the nervous and the immune system—immunoception—is receiving increasing attention, with evidence showing how immune imbalances activate specific brain regions and how neuronal regulation shapes inflammatory diseases^11–16^. One molecule that may link reduced inflammatory activity in pregnant individuals who have MS with neuronal control of peripheral immunity is GDF-15. During pregnancy, trophoblasts and immature dendritic cells in the placenta produce GDF-15, with maternal plasma concentrations rising as the fetus develops^17^. Most circulating GDF-15 originates from the fetus rather than maternal reproductive cells^18^. Low GDF-15 serum levels have been linked to miscarriage^19^, suggesting a key role in fetal immune tolerance and potentially in suppressing autoreactive CNS-directed T cell responses. Consistently, GDF-15 suppresses lymphoproliferation in systemic lupus erythematosus^20^.

Previous work identified GDNF family receptor α-like (GFRAL) as the canonical GDF-15 receptor responsible for inducing cachexia and anorexia^21,22^. GFRAL is primarily expressed in brainstem neurons^23^, and no alternative immune cell receptor has been found, despite studies reporting direct T cell modulation by GDF-15^24^, inhibition of neutrophil and macrophage chemotaxis^25,26^, and reduced cytotoxicity of tumor-infiltrating macrophages^27^, as well as GFRAL-independent modulation of myeloid cells^28^. GFRAL-expressing neurons cluster in the area postrema and to a lesser extent the nucleus tractus solitarius (NTS). Because these regions possess highly fenestrated capillaries, they are ideally positioned to sense soluble circulating mediators^29^, including GDF-15 released by the fetus or from stressed tissues^18,30^.

In this study, we explore how GDF-15 regulates autoimmune T cell responses downstream of GFRAL and demonstrate the effect of immunoception on neuroinflammation.

## Results

### GDF-15 expression is increased in human and mouse pregnancies

**Fig. 1:**
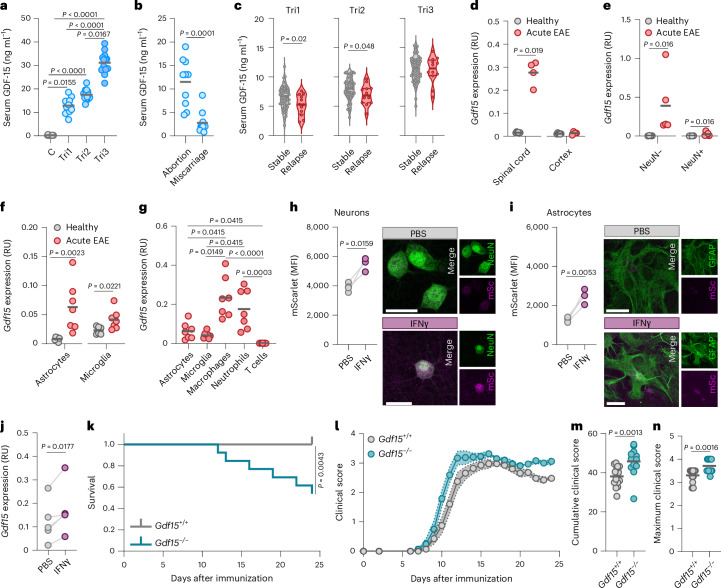
GDF-15 is induced in pregnancy and CNS inflammation. **a**, Serum levels of GDF-15 in pregnant women collected from trimester 1 (Tri1) to trimester 3 (Tri3), and age-matched and BMI-matched nonpregnant controls (C); *n* = 13. **b**, GDF-15 serum concentrations in women experiencing miscarriage and those undergoing elective abortion in Tri1; *n* = 10 per group. **c**, Serum concentrations of GDF-15 in individuals with MS throughout pregnancy stratified by stable disease (*n* = 58) versus relapse (*n* = 12) during pregnancy. **d**–**g**, *Gdf15* expression measured by quantitative PCR with reverse transcription (RT–qPCR) in **d**, spinal cord and cortex tissue of female healthy (*n* = 6) and acute EAE (day 15 post immunization (p.i.), *n* = 4) mice; **e**, spinal cord neuronal (NeuN^+^) and nonneuronal (NeuN^*−*^) nuclei of female healthy (*n* = 4) and acute EAE (day 15 p.i., *n* = 5) mice; **f**, spinal cord astrocytes and microglia of female healthy (*n* = 6) and acute EAE (day 15/16 p.i., *n* = 7) mice; and **g**, spinal cord astrocytes, microglia, macrophages, neutrophils and T cells in acute EAE (day 15/16 p.i.) mice; *n* = 7. **h**,**i**, Mean fluorescence intensity (MFI) of a P_*Gdf15*_-mScarlet reporter in neurons (**h**) and astrocytes (**i**) after stimulation with 100 ng ml^*−*1^ IFNγ for 72 h; *n* = 4. Scale bars, 20 µm. **j**, *Gdf15* expression quantified by RT–qPCR in primary neuron–astrocyte co-cultures (14 days in vitro) treated with 100 ng ml^*−*1^ IFNγ for 72 h; *n* = 5. **k**–**n**, EAE was induced in *Gdf15*^*+/+*^ (*n* = 14) and *Gdf15*^*−/−*^ (*n* = 13) mice. **k**, Survival. **l**, Mean clinical score. **m**, Cumulative clinical score. **n**, Maximum clinical score. Individual data points represent biological replicates. Data are shown as the mean ± s.e.m. (**l**). In **a** and **g**, a Kruskal–Wallis test with false discovery rate (FDR) correction was performed. In **b**, **d**–**f**, **m** and **n**, two-sided Mann–Whitney tests were performed. In **c**, a Wilcoxon test was performed. In **h**–**j**, a two-sided paired *t*-test was performed. For **k**, a log-rank Mantel–Cox test was used. RU, relative units. Source data

**Extended Data Fig. 1:**
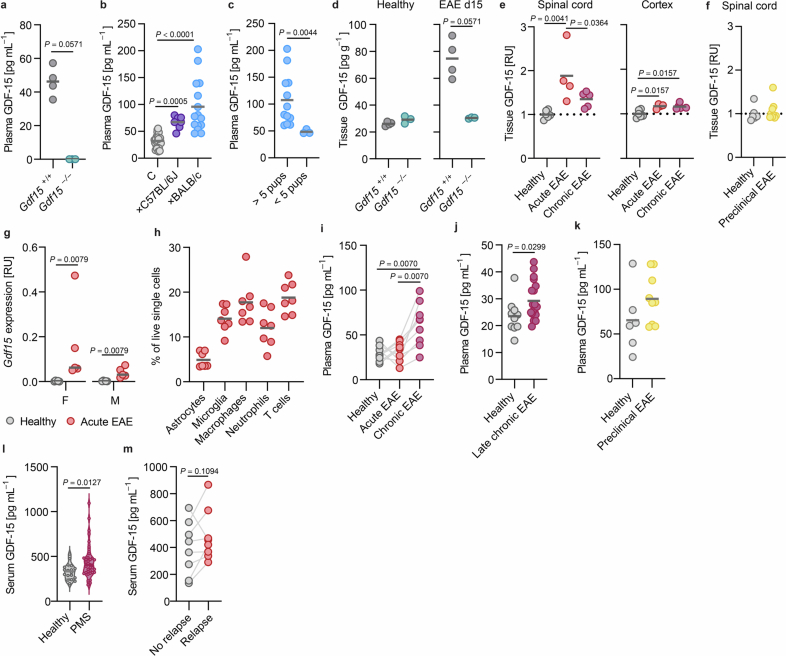
Chronic CNS inflammation elevates peripheral GDF-15 levels. **a**-**c**, Plasma concentrations of GDF-15 in a, healthy female *Gdf15*^+/+^ (*n* = 4) and *Gdf15*^*−/−*^ (*n* = 3) littermates, **b**, female C57BL/6J mice from controls (C, *n* = 23), syngeneic matings (C57BL/6J, *n* = 8), or semi-allogeneic matings (BALB/c, *n* = 15) in trimester 3 (GD17.5 ± 1), and **c**, dams from semi-allogeneic matings in trimester 3, low (*n* = 3) and regular (*n* = 12) pup counts. **d**, Protein expression of GDF-15 in spinal cord measured by tissue lysate ELISA in healthy and acute EAE (day 15 post immunization (p.i.)) female *Gdf15*^+/+^ mice and *Gdf15*^*−/−*^ littermates; *n* = 4 for *Gdf15*^+/+^, *n* = 3 for *Gdf15*^*−/−*^. **e**, **f**, Relative protein levels of GDF-15 measured by tissue lysate ELISA in **e**, spinal cord and cortex of female healthy (*n* = 6), acute EAE (day 15 p.i., *n* = 4), or chronic EAE (day 30 p.i., *n* = 5) mice, and **f**, spinal cord of female healthy (*n* = 6) and preclinical EAE (day 9 p.i., *n* = 9) mice. **g**, *Gdf15* expression from spinal cord nonneuronal (NeuN^*−*^) nuclei measured by RT-qPCR in female and male healthy and acute EAE mice (day 15/16 p.i.); *n* = 5 per group. **h**, Frequency of cell populations from Fig. 1g within viable single cells in acute EAE (day 15/16 p.i.) *n* = 7. **i**-**k**, Plasma concentrations of GDF-15 in **i**, female C57BL/6J mice before EAE induction, in the acute (day 14 p.i.) and chronic phases (day 29 p.i.) *n* = 9, **j**, late chronic EAE mice (day 59 p.i.; *n* = 19) and healthy littermate controls (*n* = 10) and **k**, preclinical EAE mice (day 9 p.i.; *n* = 9) and healthy littermate controls (*n* = 6). **l**, **m**, GDF-15 serum concentrations in **l**, progressive (PMS; *n* = 36) MS patients compared to healthy controls (*n* = 18) and **m**, relapse vs. non-relapse samples from the same MS patient; *n* = 8. Individual datapoints represent biological replicates. For (**a**, **c**, **d**, **f**, **g**, **h**, **k**, **l**) two-sided Mann-Whitney tests were performed. In (**b**, **e**), Kruskal-Wallis tests with FDR correction were performed. In (**i**), a Friedman test with FDR correction was used. For (**m**), a Wilcoxon test was used. RU = Relative units. Source data

To assess whether GDF-15 contributes to fetomaternal immune tolerance, we measured its levels in human and mouse pregnancies. As reported^31^, plasma GDF-15 gradually increased in human pregnancy compared to age-matched and body mass index-matched nonpregnant controls (Fig. 1a and Supplementary Table 1). In syngeneic C57BL/6J matings, we detected only a minor increase in systemic GDF-15 during the third trimester. In contrast, in semi-allogeneic matings, which mimic exposure to foreign antigens derived from the fetus, GDF-15 levels surged nearly fourfold (Extended Data Fig. 1a,b).

Although a study postulated that a lack of GDF-15 during human pregnancy has no impact on fetal development or pregnancy^32^, we found that lower plasma GDF-15 in pregnant mice correlated with reduced litter size (Extended Data Fig. 1c). We further observed a striking difference between women experiencing a miscarriage and those undergoing an elective abortion (Fig. 1b and Supplementary Table 2). Notably, when stratifying pregnant individuals with MS according to their relapse activity during pregnancy, which can occasionally occur despite substantial pregnancy-related immunosuppression^3,4^, we found that relapse activity was associated with reduced serum GDF-15 in early to mid-pregnancy (Fig. 1c and Supplementary Table 3). This finding suggests that pregnancy-induced GDF-15 may play a key role in mediating immunosuppression in the context of neuroinflammatory diseases.

### CNS inflammation drives local GDF-15 expression

Because GDF-15 is induced by cellular stress^30^, we investigated its regulation during CNS inflammation. In experimental autoimmune encephalomyelitis (EAE), *Gdf15* expression significantly increased during acute disease (15 days after EAE induction) in the spinal cord (Fig. 1d), the primary site of immune cell infiltration, microglia activation and neurodegeneration. Consistently, the increase in GDF-15 protein levels during acute EAE was more pronounced in the spinal cord than in the cortex (Extended Data Fig. 1d,e), whereas GDF-15 remained unchanged in the preclinical phase of the disease (Extended Data Fig. 1f). *Gdf15* transcript induction was most pronounced in nonneuronal cells, although neurons also significantly upregulated *Gdf15* expression during acute EAE (Fig. 1e and Supplementary Data Fig. 1a). This increase was similar in female and male mice (Extended Data Fig. 1g). Among nonneuronal cells, both astrocytes and microglia induced *Gdf15* expression during acute EAE (Fig. 1f and Supplementary Data Fig. 1b). However, the strongest *Gdf15* expression was detected in infiltrating myeloid cells (Fig. 1g and Extended Data Fig. 1h).

In accordance with previous reports using peripheral GDF-15 as a biomarker for neurodegenerative diseases^33,34^, plasma GDF-15 levels were elevated in the chronic phase of the disease (29 days after EAE induction; Extended Data Fig. 1i,j), with no significant increase detected before disease onset (Extended Data Fig. 1k). Individuals with progressive MS also showed significantly elevated serum GDF-15 (Extended Data Fig. 1l and Supplementary Table 4). Although individuals with acute relapse events also exhibited increased GDF-15 levels, this effect was not statistically significant (Extended Data Fig. 1m and Supplementary Table 4). Together, these results suggest that GDF-15 is upregulated in response to acute inflammation within the spinal cord and can be detected in the circulation during chronic stages of neuroinflammatory diseases such as MS, where it may contribute to disease stability or regression^35^.

To investigate which signals drive *Gdf15* expression, we generated a lentiviral GDF-15 reporter construct^36^ that includes the putative *Gdf15* promoter sequence 1.7 kb upstream of the transcription start site, followed by a reporter fluorochrome (Supplementary Data Fig. 2a). We validated the *Gdf15*-mScarlet reporter in the neuronal cell line Neuro-2a using an inducer of the unfolded protein response, tunicamycin^37^, resulting in a twofold increase in fluorescence intensity (Supplementary Data Fig. 2b). In primary neuron–astrocyte cultures, we observed a significant increase in *Gdf15* promoter activity in response to the NFE2-like bZIP transcription factor 2 (NRF2) activator 4-octyl itaconate (4-OI), which mimics downstream effects of oxidative stress, a hallmark of neuroinflammation^1^, both in neurons and astrocytes (Supplementary Data Fig. 2c). Moreover, chronic stimulation with interferon-γ (IFNγ), a cytokine released by infiltrating T cells during autoimmune neuroinflammation^2^, also increased *Gdf15* reporter expression in neurons and astrocytes (Fig. 1h,i). We corroborated this finding at the mRNA level (Fig. 1j). In microglia, *Gdf15* induction was limited to activation of Toll-like receptor 4 (TLR4) by lipopolysaccharide, but this effect was not statistically significant (Supplementary Data Fig. 2d,e). These findings suggest that upregulation of *Gdf15* in CNS-resident cells is orchestrated in a cell-type-specific manner in response to various molecular cues of cellular stress and inflammation.

### Loss of *Gdf15* exacerbates CNS inflammation

**Extended Data Fig. 2:**
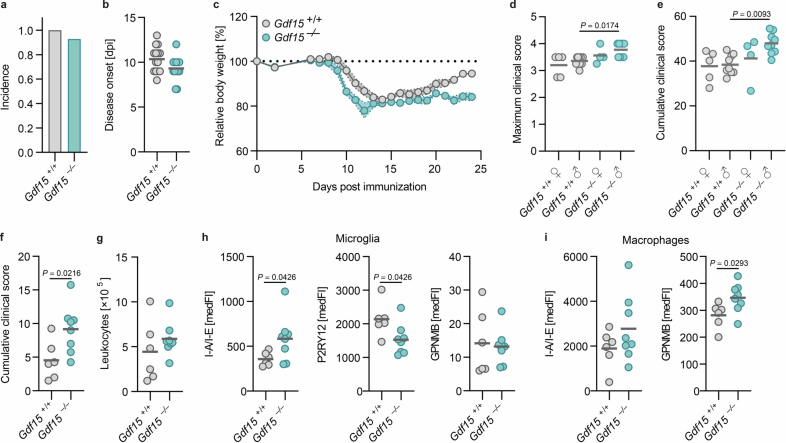
*Gdf15* deletion exacerbates CNS inflammation. **a**-**e**, EAE was induced in *Gdf15*^*+/+*^ (*n* = 14) and *Gdf15*^*−/−*^ (*n* = 13) mice. **a**, Incidence, **b**, disease onset, and **c**, relative body weight loss. **d**, Maximum clinical score and **e**, cumulative clinical score in female and male *Gdf15*^*+/+*^ and *Gdf15*^*−/−*^ mice; *n* = 9 for males, *n* = 5 for *Gdf15*^*+/+*^ females and *n* = 4 for *Gdf15*^*−/−*^ females. **f**-**i**, Characterization of microglia and CNS-infiltrating immune cells during acute EAE (day 15 p.i.); *n* = 6 for *Gdf15*^*+/+*^*, n* = 8 for *Gdf15*^*−/−*^. **f**, Cumulative clinical score. **g**, Absolute number of viable leukocytes (CD45^hi^). **h**, Median fluorescence intensity (medFI) of MHC II, P2RY12 and GPNMB on microglia. **i**, MedFI of MHC II and GPNMB on infiltrating macrophages. Individual datapoints represent biological replicates. Data are shown as mean ± s.e.m. (**c**). For (**a**), a Fisher’s exact test was used. For (**d**, **e**), a Kruskal-Wallis test with FDR correction was performed. In (**b**, **f**-**i**), a two-sided Mann-Whitney test was performed. Dpi, days post immunization. Source data

Based on previous studies^35,38,39^, we next hypothesized that GDF-15 induction during cellular stress initiates an immunoregulatory program that counteracts acute inflammation and protects neurons from inflammation-induced cell death. Although constitutive loss of *Gdf15* (ref. ^40^) did not alter EAE disease incidence or onset (Extended Data Fig. 2a,b), it significantly reduced survival (Fig. 1k) and worsened clinical outcomes during EAE (Fig. 1l–n and Extended Data Fig. 2c–e).

We found no significant difference in CNS immune cell infiltrates in *Gdf15*-deficient animals compared to control littermates during acute EAE (Extended Data Fig. 2f,g and Supplementary Data Fig. 3a). However, microglia in *Gdf15*-deficient animals exhibited increased expression of major histocompatibility complex class II and decreased expression of the purinergic receptor P2RY12 (Extended Data Fig. 2h and Supplementary Data Fig. 3b), indicating a pro-inflammatory phenotype. Additionally, we found increased expression of glycoprotein NMB (GPNMB), a phagocyte marker implicated in neurodegeneration^41^, in infiltrating macrophages (Extended Data Fig. 2i and Supplementary Data Fig. 3b). Overall, these results suggest that GDF-15 mediates containment and resolution of neuroinflammation.

### Therapeutic GDF-15 delivery protects against neuroinflammation

**Extended Data Fig. 3:**
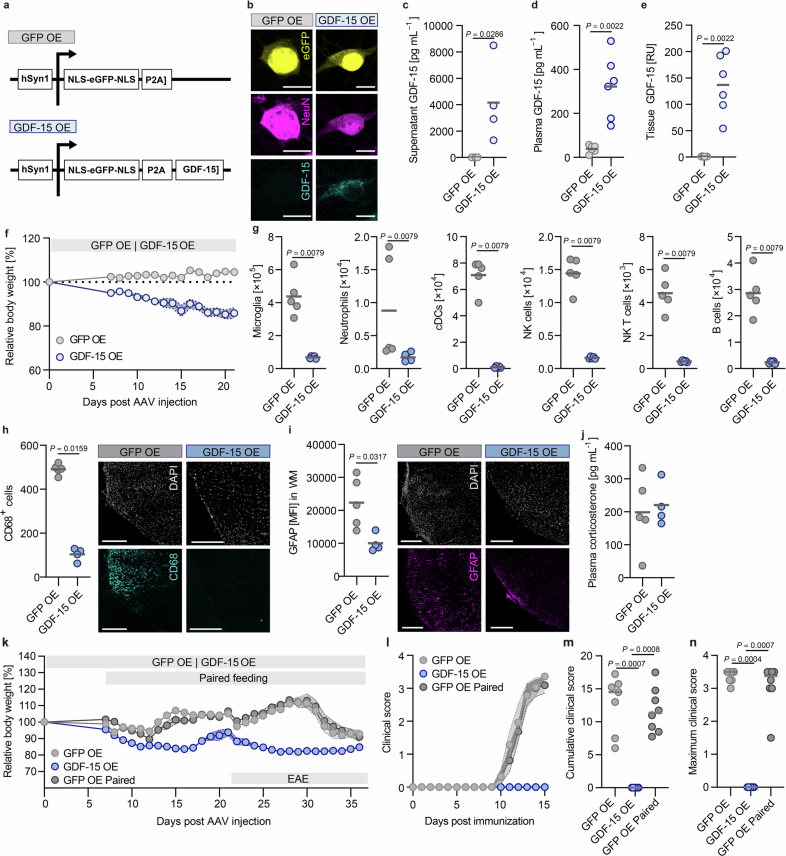
Neuronal GDF-15 delivery induces body weight loss. **a**, Gene delivery construct. hSyn1, Human synapsin 1 promoter; NLS, nuclear localization sequence; P2A, peptide 2A cleavage site; closed reading frame. **b**, Staining of GDF-15 in primary cortical neurons transduced with an rAAV coding for eGFP (GFP OE) or mouse GDF-15 (GDF-15 OE) at a multiplicity of infection (MOI) of 20,000 on DIV7. Scale bars, 10 µm. **c**, GDF-15 concentration in supernatant from transduced primary cortical neurons; *n* = 4. **d**-**f**, Female C57BL/6J mice were injected with a GFP OE or GDF-15 OE rAAV; *n* = 6 per group. **d**, Plasma concentration and **e**, brain tissue concentration of GDF-15 on day 21 post-injection. **f**, Relative body weight change. **g**-**j**, Female C57BL/6J mice were injected with a GFP OE or GDF-15 OE rAAV prior to EAE induction. Analyses were performed in acute EAE (day 15 post immunization (p.i.)). **g**, Quantification of immune cell infiltrates in the brain and spinal cord in; *n* = 5 per group. **h**, Quantification of CD68^+^ myeloid cells in cervical spinal cord sections; *n* = 5 for GFP OE, *n* = 4 for GDF-15 OE. Scale bars, 200 µm. **i**, Mean fluorescence intensity (MFI) of GFAP staining in the white matter (WM) of cervical spinal cord sections; *n* = 5 for GFP OE, *n* = 4 for GDF-15 OE. Scale bars, 200 µm. **j**, Plasma concentrations of corticosterone; *n* = 5 for GFP OE, *n* = 4 for GDF-15 OE. **k**-**n**, Female C57BL/6J mice were injected with a GFP OE or GDF-15 OE rAAV; *n* = 7 for GFP OE and GDF-15 OE, *n* = 8 for GFP OE Paired. **k**, Relative body weight loss, **l**, mean clinical disease score, **m**, cumulative disease score, and **n**, maximum disease score after EAE injection. Individual datapoints represent biological replicates. Data are shown as mean ± s.e.m. (**f**, **k**, **l**). For (**c**-**e**, **g**-**j**), two-sided Mann-Whitney tests were performed. For (**m**, **n**), a Kruskal-Wallis test with FDR correction was performed. OE, overexpression; RU, relative units. Source data

We next aimed to therapeutically harness GDF-15 to mitigate CNS inflammation. We developed a construct for direct mouse GDF-15 delivery to neurons using the human synapsin 1 promoter (Extended Data Fig. 3a). Transduction of primary neurons with a recombinant adeno-associated virus (rAAV) harboring this construct resulted in robust GDF-15 expression in neurons (Extended Data Fig. 3b) and secretion (Extended Data Fig. 3c). Using a low rAAV titer, intravenous injections for neuronal transduction achieved physiological plasma concentrations similar to those observed during late mouse pregnancy (Extended Data Fig. 3d) and high CNS expression (Extended Data Fig. 3e). As anticipated, due to GFRAL engagement and activation of anorexic effects^21,22^, mice lost approximately 10% of body weight following neuronal GDF-15 delivery (Extended Data Fig. 3f).

**Fig. 2:**
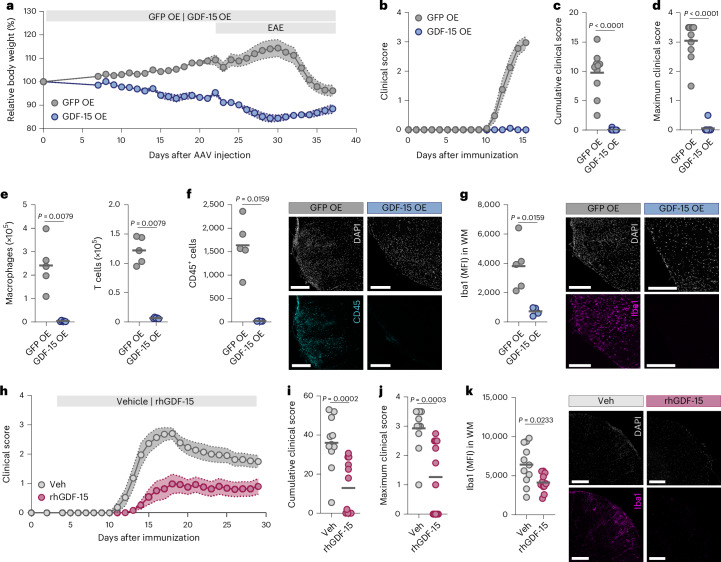
GDF-15 delivery is protective in neuroinflammation. **a**, Relative body weight change after injecting female C57BL/6J mice with an rAAV encoding eGFP (GFP OE, *n* = 10) or mouse GDF-15 (GDF-15 OE, *n* = 9). **b**, Mean clinical disease score. **c**, Cumulative disease score. **d**, Maximum disease score after immunization with MOG_35–55_ 3 weeks after rAAV injection; *n* = 10 for GFP OE, *n* = 9 for GDF-15 OE. **e**, Absolute numbers of macrophages and T cells in brain and spinal cord tissue quantified by flow cytometry in acute EAE (day 15 p.i.); *n* = 5 per group. **f**, Quantification of infiltrating CD45^+^ cells in cervical spinal cord sections in acute EAE (day 15 p.i.); *n* = 5 for GFP OE, *n* = 4 for GDF-15 OE. Scale bars, 200 µm. **g**, Mean fluorescence intensity (MFI) of Iba1 staining in the white matter (WM) of cervical spinal cord sections in acute EAE; *n* = 5 for GFP OE, *n* = 4 for GDF-15 OE. Scale bars, 200 µm. **h**–**k**, Female C57BL/6J mice were immunized with MOG_35–55_ and received daily subcutaneous injections of 5 nmol per kg rhGDF-15 (*n* = 12) or a vehicle control (Veh, *n* = 11) from day 4 p.i. onward. **h**, Mean clinical score. **i**, Cumulative disease score. **j**, Maximum disease score. **k**, MFI of Iba1 staining in the WM of cervical spinal cord sections in chronic EAE (day 29 p.i.); *n* = 11 per group. Scale bars, 200 µm. Individual data points represent biological replicates. Data are shown as the mean ± s.e.m. (**a**, **b** and **h**). For **c**–**g** and **i**–**k**, Mann–Whitney tests were performed. OE, overexpression. Source data

In metabolic cages, these mice showed reduced food intake, energy expenditure and respiratory exchange ratio (Supplementary Data Fig. 4a–c), consistent with prior studies using recombinant GDF-15^42^. Notably, rAAV-mediated *Gdf15* delivery to the CNS completely prevented neuroinflammation (Fig. 2a–d and Supplementary Data Fig. 5a) and we detected only a small number of CNS-infiltrating immune cells (Fig. 2e,f, Extended Data Fig. 3g and Supplementary Data Fig. 5b). This was accompanied by significantly reduced Iba1 and GFAP immunoreactivity, as well as lower numbers of CD68^+^ myeloid cells in spinal cord sections from acute EAE animals (Fig. 2g, Extended Data Fig. 3h,i and Supplementary Data Fig. 5c,d). Together, inflammatory signals from both CNS-infiltrating and CNS-resident cells were diminished. Importantly, engagement of GFRAL by GDF-15 did not activate the hypothalamic–pituitary–adrenal axis^43^, as plasma corticosterone remained unchanged (Extended Data Fig. 3j), suggesting that protection from neuroinflammation was not driven by corticosteroid-mediated immunosuppression.

Because GDF-15 reduces appetite via GFRAL^21,22^ and we observed body weight loss after rAAV-mediated *Gdf15* delivery to the CNS (Fig. 2a and Extended Data Fig. 3f), we performed paired-feeding experiments to exclude caloric restriction as a confounder. While mice typically receive food ad libitum, an additional group with neuronal GFP overexpression (GFP OE) was pair-fed, receiving restricted amounts of food based on the intake of the GDF-15-overexpressing (GDF-15 OE) mice (Supplementary Fig. 6a). Despite caloric restriction, body weight loss in the pair-fed group was minimal (Supplementary Data Fig. 6b,c). In GDF-15 OE mice, weight loss was partly due to reduced fat mass, which was not observed in pair-fed GFP OE mice (Supplementary Data Fig. 6d). Importantly, pair-fed mice were not protected from neuroinflammation with similar disease severity and immune cell infiltration than GFP OE mice with ad libitum food access (Extended Data Fig. 3k–n and Supplementary Data Fig. 6e,f). Thus, caloric restriction alone does not account for GDF-15-mediated protection from neuroinflammation.

**Extended Data Fig. 4:**
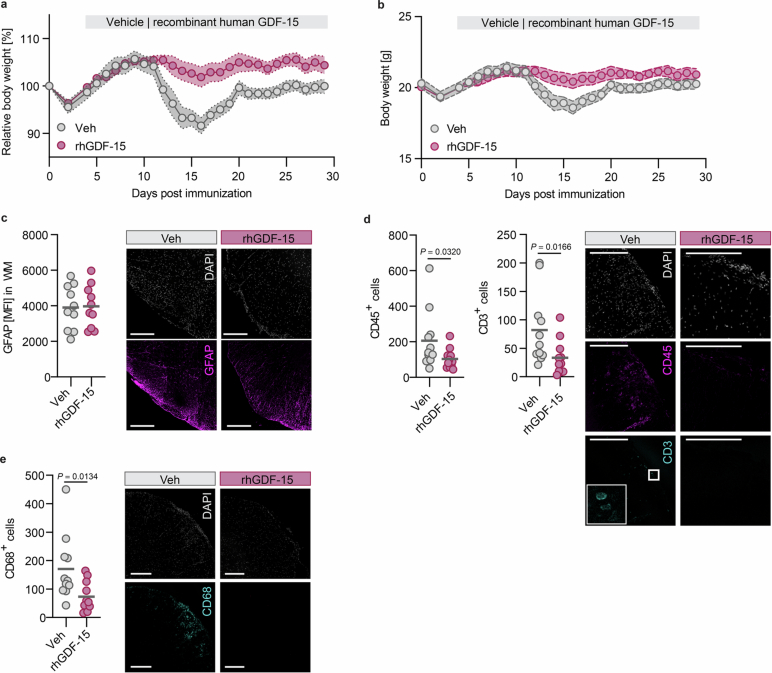
Recombinant GDF-15 prevents immune cell infiltration. EAE was induced in female C57BL/6J mice, and animals received daily subcutaneous injections of 5 nmol per kg of body weight of recombinant human GDF-15 (rhGDF-15, *n* = 12) or a vehicle control (Veh, *n* = 11) from day 4 post immunization (p.i.) onwards. **a**, Relative body weight loss. **b**, Absolute body weight. **c**, Mean fluorescence intensity (MFI) of GFAP staining in the white matter (WM) of cervical spinal cord sections in chronic EAE (day 29 p.i.); *n* = 10 per group. Scale bars, 200 µm. **d**, Number of leukocytes (CD45^+^) and T cells (CD3^+^), and **e**, myeloid cells (CD68^+^) in cervical spinal cord sections in chronic EAE (day 29 p.i.); *n* = 11 per group. Scale bars, 200 µm. Individual datapoints represent biological replicates. Data are shown as mean ± s.e.m. (**a**, **b**). For (**c**-**e**), two-sided Mann-Whitney test were performed. Source data

To evaluate clinical translatability, EAE mice received daily subcutaneous recombinant human GDF-15 (rhGDF-15; 5 nmol per kg of body weight)^22,44^. Although this medium dose did not induce body weight loss (Extended Data Fig. 4a,b), it significantly reduced clinical disability and microglia activation, as indicated by decreased Iba1 reactivity in spinal cord white matter (Fig. 2h–k). We observed no differences in GFAP immunoreactivity, but fewer infiltrating leukocytes, T cells and CD68^+^ myeloid cells (Extended Data Fig. 4c–e). In conclusion, both gene therapy and rhGDF-15 treatment were highly effective in preventing immune cell infiltration into the CNS, thereby protecting from neuroinflammation.

### GDF-15 delivery suppresses CD4^+^ T cell activation

**Fig. 3:**
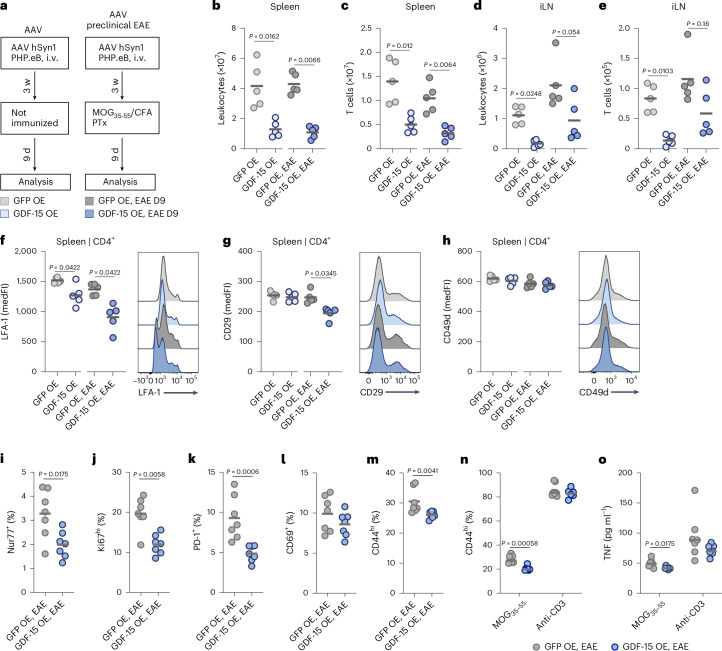
GDF-15 delivery suppresses peripheral CD4^+^ T cell activation. Female C57BL/6J mice were injected with an rAAV encoding eGFP (GFP OE) or mouse GDF-15 (GDF-15 OE). In preclinical EAE groups, mice were immunized and organs were excised on day 9 p.i. before disease onset. **a**, Timeline for rAAV injection and EAE induction. i.v., intravenous. **b**–**e**, Absolute numbers quantified by flow cytometry; *n* = 5 per group. **b**, Leukocytes in the spleen. **c**, T cells in the spleen. **d**, Leukocytes in the inguinal lymph nodes (iLNs). **e**, T cells in the iLNs. **f**–**h**, Median fluorescence intensity (medFI) of selected markers on CD4^+^ T cells in the spleen analyzed by flow cytometry; *n* = 5 per group. **f**, LFA-1. **g**, CD29. **h**, CD49d. **i**–**m**, Frequency of selected population within CD4^+^ T cells in the spleen 9 days p.i. analyzed by flow cytometry; *n* = 7 per group. **i**, Nur77. **j**, Ki67. **k**, PD-1. **l**, CD69. **m**, CD44. **n**,**o**, Splenocytes were isolated 9 days p.i. and restimulated with 5 µg ml^*−*1^ MOG_35–55_ or 0.5 µg ml^*−*1^ anti-CD3 as a positive control; *n* = 7 per group. **n**, Frequency of CD44^hi^ cells within total CD4^+^ T cells after 72 h. **o**, Concentration of tumor necrosis factor (TNF) in supernatant after 48 h. Individual data points represent biological replicates. For **b**–**o**, two-sided Mann–Whitney tests were used. OE, overexpression. Source data

We next sought to elucidate how GDF-15 impacts the recruitment of peripheral immune cells to the CNS. Following GDF-15 delivery to the CNS, we analyzed the peripheral immune cell compartments in preclinical EAE mice (9 days after immunization) and in healthy controls (Fig. 3a and Supplementary Data Fig. 7a). We detected a decrease in CD45^+^ leukocytes, T cells and B cells in the spleen and inguinal lymph nodes (iLNs) in response to increased GDF-15 in both EAE mice and healthy controls (Fig. 3b–e and Supplementary Data Fig. 7b–d).

**Extended Data Fig. 5:**
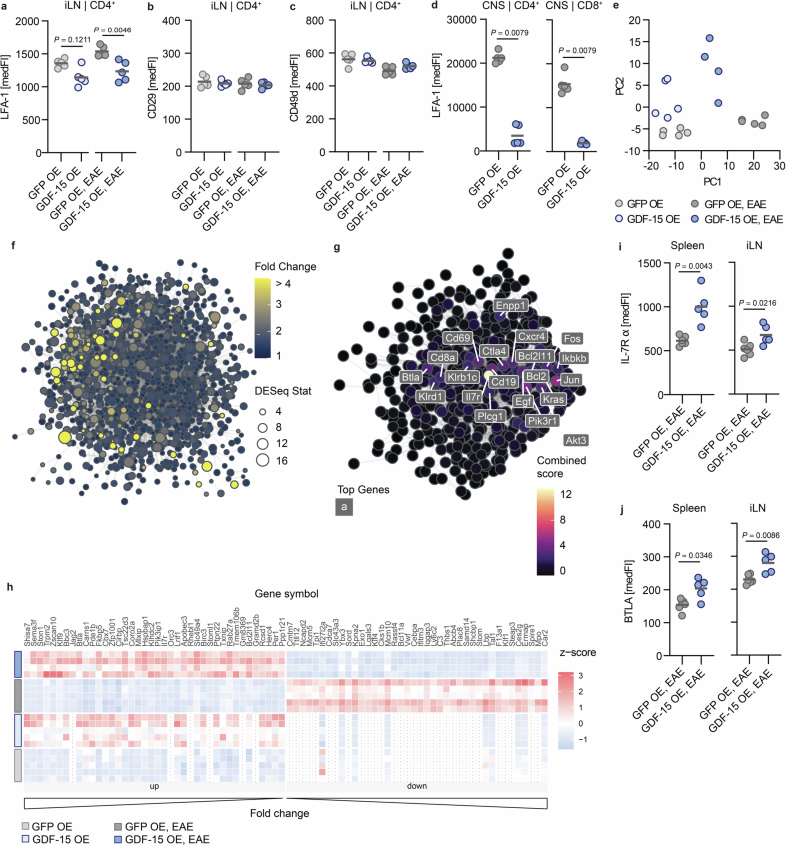
GDF-15 delivery modulates CD4^+^ T cell phenotype. Female C57BL/6J mice were injected with an rAAV coding for eGFP (GFP OE) or mouse GDF-15 (GDF-15 OE). In preclinical EAE groups, mice were immunized, and organs were collected on day 9 post immunization (p.i.) prior to disease onset. **a**-**c**, Median fluorescence intensity (medFI) of selected markers on CD4^+^ T cells in the iLN; *n* = 5 per group. **d**, MedFI of LFA-1 on CD4^+^ and CD8^+^ T cells in the CNS during acute EAE (day 15 p.i.); *n* = 5 per group. **e**-**h**, Splenic CD4^+^ T cells were purified and processed for mRNA sequencing; *n* = 5 per group. **e**, Principal component analysis. **f**, Fold change and statistics for KEGG pathway analysis between GFP OE, EAE and GDF-15 OE; EAE group. **g**, Top regulated genes in upper left cluster. **h**, Heatmap of differentially regulated genes. **i**, **j**, medFI of selected markers on CD4^+^ T cells three weeks after rAAV injection on day 9 p.i.; *n* = 5 per group. **i**, Interleukin (IL)-7Rα in the spleen and inguinal lymph nodes (iLN). **j**, B lymphocyte and T lymphocyte attenuator (BTLA) in the spleen and iLN. Individual datapoints represent biological replicates. For (**a**-**d**, **i**, **j**), two-sided Mann-Whitney tests were performed. OE, overexpression. Source data

We next assessed integrins required for leukocyte rolling on endothelial cells^45^ and T cell transmigration across the blood–brain barrier^2^, specifically LFA-1 and VLA-4, the latter consisting of subunits CD29 and CD49d. We found that GDF-15 consistently downregulated the active, high-affinity conformation of LFA-1 on splenic (Fig. 3f) and iLN (Extended Data Fig. 5a) CD4^+^ T cells, with weaker modulation of CD29 and CD49d (Fig. 3g,h and Extended Data Fig. 5b,c). The effect on LFA-1 expression was even more pronounced after EAE immunization. A similar pattern was observed for CD8^+^ T cells (Supplementary Data Fig. 7e–g). Notably, downregulation of LFA-1 on CD4^+^ and CD8^+^ T cells was also evident in the CNS during acute EAE (Extended Data Fig. 5d).

To identify additional effector molecules beyond integrins, we isolated splenic CD4^+^ T cells from the same experimental groups for bulk RNA sequencing (Extended Data Fig. 5e). We found that several checkpoint molecules increased expression following GDF-15 delivery, with *Il7r* identified as the top upregulated gene (Extended Data Fig. 5f–h). We validated increased protein expression of interleukin (IL)-7Rα—an exclusion marker for T cell activation^46^—and the inhibitory co-receptor B lymphocyte and T lymphocyte attenuator on splenic and iLN CD4^+^ cells (Extended Data Fig. 5i,j).

Moreover, in splenic CD4^+^ T cells during preclinical EAE (Supplementary Data Fig. 7h,i), GDF-15 delivery led to a pronounced downregulation of the immediate early activation marker Nur77 (Fig. 3i), the proliferation marker Ki67 (Fig. 3j) and additional indicators of T cell activation and memory formation, including programmed death-1 (PD-1), CD69 and CD44 (Fig. 3k–m). Restimulation of T cells from GDF-15 OE mice with MOG_35–55_ peptide resulted in reduced memory T cell formation (Fig. 3n) and diminished secretion of tumor necrosis factor (Fig. 3o).

Together, elevation of GDF-15 not only reduces the overall T cell pool but also induces co-inhibitory molecules while suppressing LFA-1 expression, antigen-specific activation and proliferation, thereby limiting T cell transmigration into the CNS and pro-inflammatory functions.

### GDF-15 delivery activates β-adrenergic signaling in the spleen

**Fig. 4:**
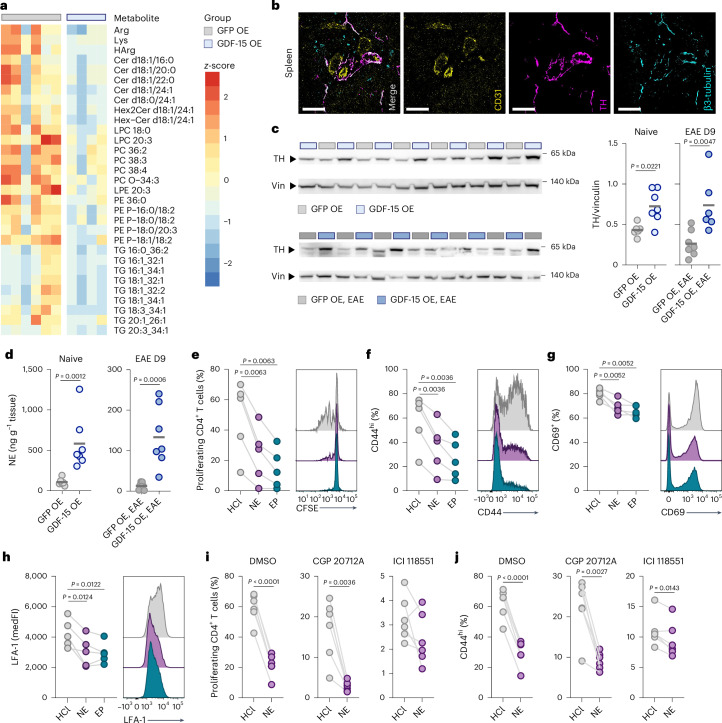
GDF-15 delivery activates β-adrenergic signaling in the spleen. **a**, Female C57BL/6J mice were injected with an rAAV encoding eGFP (GFP OE) or mouse GDF-15 (GDF-15 OE). Plasma was collected 3 weeks after rAAV injection for liquid chromatography–mass spectrometry analysis; *n* = 6 for GFP OE, *n* = 4 for GDF-15 OE. LPC, lysophosphatidylcholine; LPE, lysophosphatidylethanolamine; PC, phosphatidylcholine; PE, phosphatidylethanolamine; TG, triglyceride. **b**, Expression of tyrosine hydroxylase (TH) in spleen-innervating neurons surrounded by blood vessels. Scale bars, 100 µm. **c**,**d**, Female C57BL/6J mice were injected with an rAAV encoding GFP OE or GDF-15 OE. For EAE mice, organs were excised on day 9 p.i. **c**, Immunoblot for TH in the spleen of naive or preclinical EAE mice. Expression was normalized to vinculin (Vin). Naive: *n* = 6 for GFP OE, *n* = 7 for GDF-15 OE; EAE day 9: *n* = 7 for GFP OE, *n* = 6 for GDF-15 OE. **d**, Norepinephrine (NE) concentration in the spleens of naive or preclinical EAE mice normalized to tissue weight. Naive: *n* = 6 for GFP OE, *n* = 7 for GDF-15 OE; EAE day 9: *n* = 7 for GFP OE, *n* = 7 for GDF-15 OE**. e**–**h**, Primary mouse T cells were stimulated with anti-CD3/CD28 and 10 µM NE, 10 µM epinephrine (EP) or hydrochloric acid (HCl) as vehicle control; *n* = 5. **e**,**f**, Proliferation measured by CFSE dye dilution (**e**) and frequency of CD44^hi^ cells (**f**) within total CD4^+^ T cells after 72 h. **g**,**h**, Frequency of CD69^+^ cells (**g**) and medFI of LFA-1 (**h**) in total CD4^+^ T cells after 24 h. **i**,**j**, Primary mouse T cells were stimulated with anti-CD3/CD28 and 10 µM CGP 20712A, 10 µM ICI 118551 or dimethylsulfoxide (DMSO) as vehicle control before treatment with 10 µM NE or HCl; *n* = 6. **i**,**j**, Proliferation measured by CFSE dye dilution (**i**) and frequency of CD44^hi^ cells (**j**) within total CD4^+^ T cells after 72 h. Individual data points represent biological replicates. In **b**, data from one representative animal is shown. For **c** and **d**, two-sided Mann–Whitney tests were performed. For **e**–**h**, a paired one-way analysis of variance (ANOVA) with HCl as control group and FDR correction was performed. For **i** and **j**, paired two-sided *t*-tests were used. OE, overexpression. Source data

**Extended Data Fig. 6:**
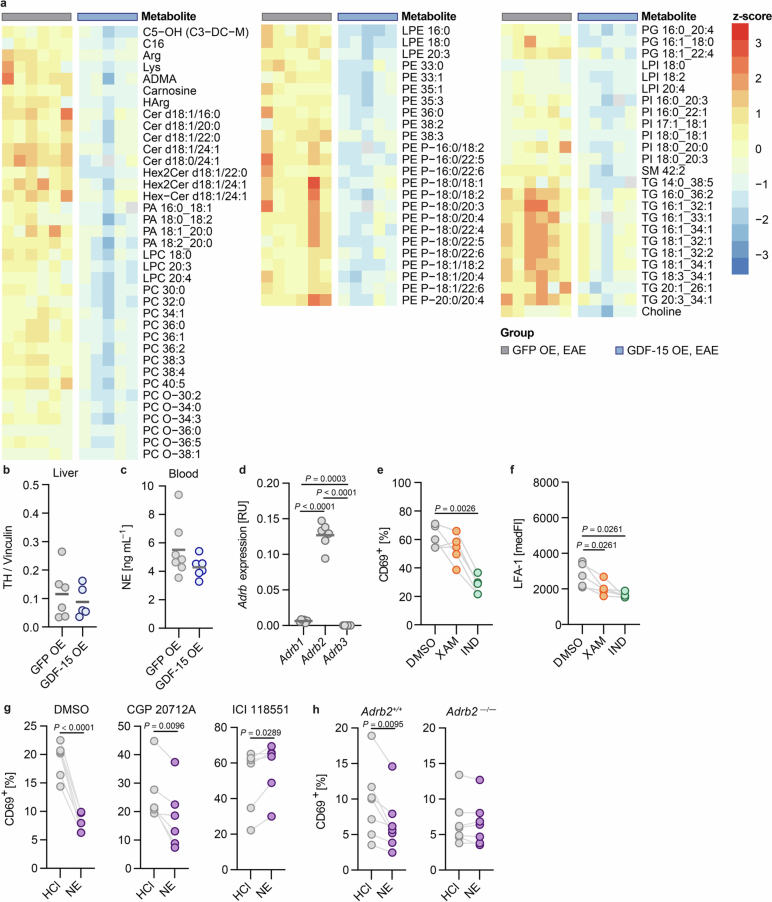
Selective activation of β2-adrenoceptors inhibits T cell activation. **a**-**c**, Female C57BL/6J mice were injected with an rAAV coding for eGFP (GFP OE, *n* = 6) or mouse GDF-15 (GDF-15 OE, *n* = 5). **a**, Metabolomics of plasma collected in the preclinical EAE phase (9 days post immunization, p.i.); *n* = 6 for GFP OE, *n* = 5 for GDF-15 OE. LPC, lysophosphatidylcholine; LPE, lysophosphatidylethanolamine; LPI, lysophosphatidylinositol; PA, phosphatidic acid; PC, phosphatidylcholine; PE, phosphatidylethanolamine; PI, phosphatidylinositol; SM, sphingomyelin; TG, triglyceride. **b**, Quantification of tyrosine hydroxylase (TH) in the liver of rAAV-injected mice by immunoblot. Expression was normalized to vinculin (Vin); *n* = 6 for GFP OE, *n* = 5 for GDF-15 OE. **c**, Blood norepinephrine (NE) concentration in naive female mice with (GFP OE, *n* = 7) or (GDF-15 OE, *n* = 6) three weeks after rAAV injection. **d**, *Adrb1/2/3* expression in CD4^+^ T cells from female mice measured by RT-qPCR; *n* = 6. **e**, **f**, Primary mouse T cells were stimulated with anti-CD3/CD28 and 10 µM xamoterol (XAM), 10 µM indacaterol (IND) or DMSO; *n* = 5. **e**, Frequency of CD69^+^ cells and **f**, median fluorescence intensity (medFI) of LFA-1 on total CD4^+^ T cells stimulated after 24 h. **g**, Primary mouse T cells were stimulated with anti-CD3/CD28 and 10 µM CGP 20712A, 10 µM ICI 118551, or DMSO as vehicle control prior to treatment with 10 µM NE or HCl. Frequency of CD69^+^ cells within total CD4^+^ T cells after 24 h; *n* = 6. **h**, Primary T cells from *Adrb2*^*+/+*^ and *Adrb2*^*−/−*^ mice were stimulated with anti-CD3/CD28 and 10 µM NE or HCl. Frequency of CD69^+^ cells within total CD4^+^ T cells after 24 h; *n* = 7 per group. Individual datapoints represent biological replicates. For (**b**, **c**) an unpaired two-sided Mann-Whitney test was performed. For (**d**-**f**), a paired one-way ANOVA with FDR correction was performed. For (**g**,**h**) paired two-sided *t*-tests were performed. OE, overexpression. Source data

To explore how metabolic adaptation to GDF-15 delivery shapes autoimmune T cell responses, we performed plasma metabolomics. GDF-15 delivery to the CNS reduced systemic triglycerides, phosphatidylcholine and phosphatidylethanolamine species (Fig. 4a and Supplementary Table 5), with stronger effects following EAE induction (Extended Data Fig. 6a and Supplementary Table 6). This profile suggests increased lipolysis and decreased triglyceride synthesis, potentially resulting from sympathetic activation of adipose tissue^47,48^.

Because GDF-15 has been linked to β-adrenergic signaling^44^, we examined the expression of tyrosine hydroxylase (TH), the rate-limiting enzyme in catecholamine synthesis. Within the spleen, we detected TH^+^ nerve fibers in close proximity to blood vessels (Fig. 4b) and GDF-15 delivery to the CNS nearly doubled TH expression in the spleen in naive and preclinical EAE mice (Fig. 4c), without affecting hepatic TH expression (Extended Data Fig. 6b). Based on a publication linking splenic sympathetic innervation to T cell exhaustion^49^, we hypothesized that increased splenic TH expression after GDF-15 delivery might suppress autoimmune T cell responses through the release of norepinephrine or epinephrine. Consistent with this, we detected a local increase of splenic norepinephrine after GDF-15 delivery (Fig. 4d), while plasma norepinephrine remained unchanged (Extended Data Fig. 6c).

Indeed, treating primary mouse T cells with norepinephrine or epinephrine during T cell antigen receptor engagement significantly impaired proliferation and reduced the expression of the memory marker CD44 in CD4^+^ T cells (Fig. 4e,f and Supplementary Data Fig. 8a). Moreover, CD69 expression and LFA-1 integrin levels were significantly decreased in CD4^+^ T cells (Fig. 4g,h), whereas CD8^+^ T cells were unaffected (Supplementary Data Fig. 8b,c). CD4^+^ T cells expressed high levels of the β_2_-adrenergic receptor (*Adrb2*), while the β_1_-adrenergic receptor (*Adrb1*) was detected only at low levels (Extended Data Fig. 6d). Accordingly, selective activation of β_1_-adrenergic or β_2_-adrenergic receptors with the pharmacological agonists xamoterol or indacaterol revealed that β2-adrenergic receptor stimulation was more effective in suppressing CD4^+^ T cell activation and LFA-1 expression (Extended Data Fig. 6e,f). Similarly, pretreatment of CD4^+^ T cells with the β_2_-adrenoreceptor antagonist ICI 118551 alleviated the suppressive effects of norepinephrine. In contrast, the β_1_-adrenoreceptor antagonist CGP 20712A had little impact (Fig. 4i,j and Extended Data Fig. 6g). Genetic deletion of *Adrb2* in T cells completely abolished norepinephrine-induced downregulation of CD69 (Extended Data Fig. 6h).

Together, our findings suggest that GDF-15 engages the sympathetic nervous system to activate β_2_-adrenergic signaling in splenic CD4^+^ T cells, thereby suppressing autoimmune T cell responses.

### GDF-15-mediated protection from neuroinflammation is GFRAL dependent

**Fig. 5:**
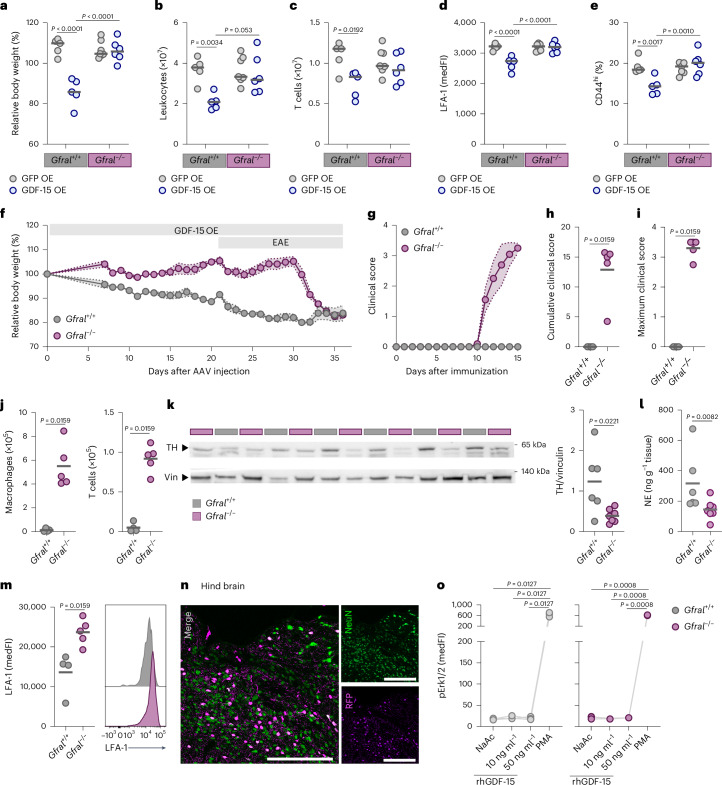
GDF-15-mediated protection from neuroinflammation is GFRAL dependent. **a**–**e**, Female mice were injected with an rAAV encoding eGFP (GFP OE) or mouse GDF-15 (GDF-15 OE); *n* = 5 for *Gfral*^*+/+*^ GFP OE, *n* = 5 for *Gfral*^*+/+*^ GDF-15 OE, *n* = 7 for *Gfral*^*−/−*^ GFP OE, *n* = 6 for *Gfral*^*−/−*^ GDF-15 OE. **a**, Relative body weight change 3 weeks after rAAV injection. **b**,**c**, Absolute number of splenic CD45^+^ leukocytes (**b**) and T cells (**c**). **d**, MedFI of LFA-1 on splenic CD4^+^ T cells. **e**, Frequency of CD44^hi^ CD4^+^ T cells in the spleen. **f**–**j**, Female *Gfral*-proficient and *Gfral*-deficient mice were injected with a GDF-15 OE rAAV and EAE was induced 3 weeks later; *n* = 4 for *Gfral*^*+/+*^, *n* = 5 for *Gfral*^*−/−*^. **f**, Relative body weight changes. **g**, Mean clinical score. **h**, Cumulative clinical score. **i**, Maximum clinical score. **j**, Absolute number of immune cells in spinal cord and brain tissue quantified by flow cytometry on day 15 p.i. **k**, Immunoblot for TH in the spleen of female GDF-15 OE mice on day 9 p.i. Expression was normalized to vinculin (Vin); *n* = 6 for *Gfral*^*+/+*^, *n* = 7 for *Gfral*^*−/−*^. **l**, Norepinephrine (NE) concentration in the spleens of male and female GDF-15 OE mice normalized to tissue weight; *n* = 6 for *Gfral*^*+/+*^, *n* = 7 for *Gfral*^*−/−*^. **m**, MedFI of LFA-1 on CD4^+^ T cells in the CNS during acute EAE (day 15 p.i.); *n* = 4 for *Gfral*^*+/+*^, *n* = 5 for *Gfral*^*−/−*^. **n**, Expression of tdTomato in the area postrema and NTS in GFRAL-Cre × Ai14 mice. Scale bars, 200 µm. **o**, Splenocytes from *Gfral*^*+/+*^ and *Gfral*^*−/−*^ mice were stimulated with increasing doses of recombinant human GDF-15 (rhGDF-15), 100 ng ml^*−*1^ phorbol 12-myristate 13-acetate (PMA) or sodium acetate (NaAc) as a vehicle control for 30 min. MedFI of pErk1/pErk2 was determined in total leukocytes (CD45^+^); *n* = 3 per group. Individual data points represent biological replicates. Data are shown as the mean ± s.e.m. (**f** and **g**). In **n**, data from one representative animal are shown. In **a**–**e**, two-way ANOVA with FDR correction was performed. For **g**–**m**, two-sided Mann–Whitney tests were performed. Data in **o** were analyzed with a paired one-way ANOVA with FDR correction. OE, overexpression. Source data

**Extended Data Fig. 7:**
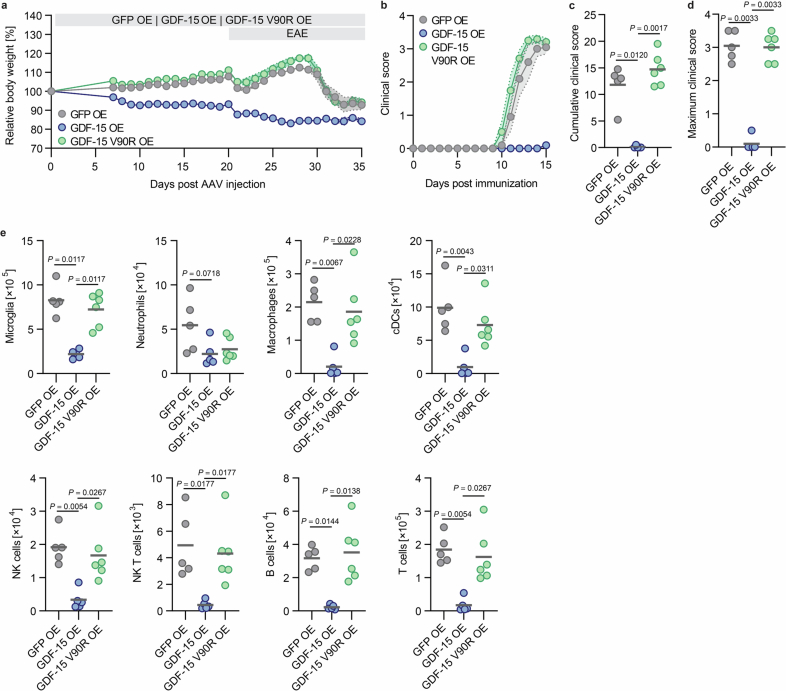
Loss of GFRAL binding activity abolishes protection of therapeutic GDF-15 delivery. Female C57BL/6J mice were intravenously injected with an rAAV coding for eGFP (GFP OE, *n* = 5), mouse GDF-15 (GDF-15 OE, *n* = 5) or mouse mutant GDF-15 p.Val90Arg (GDF-15 V90R OE, *n* = 6). **a**, Relative body weight change, **b**, mean clinical disease score, **c**, cumulative clinical disease score and **d**, maximum clinical disease score. **e**, Absolute number of microglia and infiltrating immune cells in spinal cord and brain tissue analyzed by flow cytometry on day 15 post immunization. Individual datapoints represent biological replicates. Data are shown as mean ± s.e.m. (**a**, **b**). For (**c**-**e**), a Kruskal-Wallis test with FDR correction was performed. OE, overexpression. Source data

**Extended Data Fig. 8:**
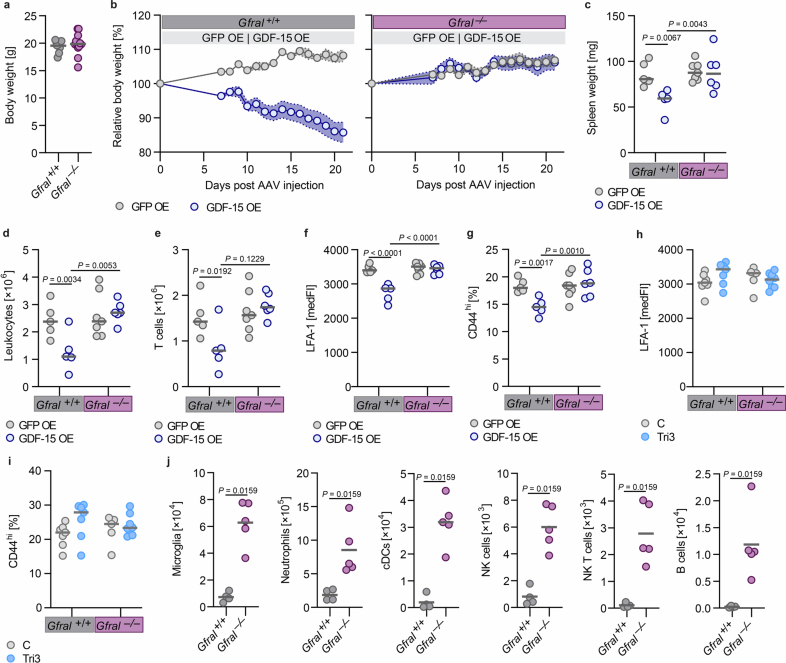
GDF-15-mediated T cell modulation is GFRAL-dependent. **a**, Baseline body weight in healthy female *Gfral*^*−/−*^ (*n* = 13) and *Gfral*^*+/+*^ (*n* = 10) mice. **b**-**g**, Female mice were injected with an rAAV coding for eGFP (GFP OE) or for mouse GDF-15 (GDF-15 OE); *n* = 5 for *Gfral*^*+/+*^ GFP OE, *n* = 5 for *Gfral*^*+/+*^ GDF-15 OE, *n* = 7 for *Gfral*^*−/−*^ GFP OE, *n* = 6 for *Gfral*^*−/−*^ GDF-15 OE. **b**, Relative body weight change. **c**, Spleen weight three weeks after rAAV injection. **d**, Absolute number of CD45^+^ leukocytes in the inguinal lymph nodes (iLNs). **e**, Absolute number of T cells in the iLNs. **f**, Median fluorescence intensity (medFI) of LFA-1 on splenic CD8^+^ T cells. **g**, Frequency of CD44^hi^ CD8^+^ T cells in the spleen. **h**-**i**, Semi-allogeneic pregnancy was induced in *Gfral*^*+/+*^ and *Gfral*^*−/−*^ mice. The expression of selected markers on splenic CD4^+^ T cells was analyzed in trimester 3 (Tri3; *n* = 7 for *Gfral*^*+/+*^ and *n* = 8 for *Gfral*^*−/−*^) animals or non-pregnant littermate controls (C; *n* = 7 for *Gfral*^*+/+*^ and *n* = 5 for *Gfral*^*−/−*^). **h**, MedFI of LFA-1, **i**, frequency of CD44^hi^ cells. **j**, Female *Gfral*^*+/+*^ (*n* = 4) and *Gfral*^*−/−*^ (*n* = 5) mice were injected with an GDF-15 OE rAAV and EAE was induced three weeks later. Quantification of infiltrating immune cells in spinal cord and brain tissue on day 15 post immunization. Individual datapoints represent biological replicates. Data are shown as mean ± s.e.m. (**b**). In (**a**, **j**) two-sided Mann-Whitney tests were performed. In (**c**-**i**) two-way ANOVA with FDR correction was used. OE, overexpression. Source data

To determine whether GFRAL mediates immunosuppression in response to neuronal GDF-15 delivery, we generated a mutant version of mouse *Gdf15* that encodes an amino acid modification analogous to the p.Val87Arg mutation in human GDF-15, which is unable to bind to GFRAL^22^. Unlike native GDF-15, this mutated version abolished the weight loss associated with neuron-restricted expression (Extended Data Fig. 7a) and negated protection from neuroinflammation (Extended Data Fig. 7b–d). The immune cell infiltrate in the CNS of mice receiving GDF-15 p.Val90Arg resembled that of GFP controls (Extended Data Fig. 7e). We also administered the GDF-15 OE rAAV to *Gfral*-deficient mice^50^, which showed no baseline deficits in body weight (Extended Data Fig. 8a). Consistent with our findings using the mouse GDF-15 p.Val90Arg, mice lacking *Gfral* expression did not lose body weight after neuronal GDF-15 delivery (Fig. 5a and Extended Data Fig. 8b). Furthermore, a significant reduction in spleen weight (Extended Data Fig. 8c), along with diminished total leukocytes and T cells in the spleen in response to GDF-15, was observed only in *Gfral*-proficient mice (Fig. 5b,c). This observation was replicated in the iLNs (Extended Data Fig. 8d,e), further corroborating that GDF-15-mediated modulation of the peripheral immune response is GFRAL dependent. We also validated that the downmodulation of LFA-1 on splenic CD4^+^ T cells (Fig. 5d) and CD8^+^ T cells (Extended Data Fig. 8f) is GFRAL dependent. Moreover, we demonstrated that GDF-15 delivery to the CNS reduces the frequency of effector memory CD4^+^ T cells (Fig. 5e) and CD8^+^ T cells (Extended Data Fig. 8g) only in *Gfral*-proficient mice.

**Extended Data Fig. 9:**
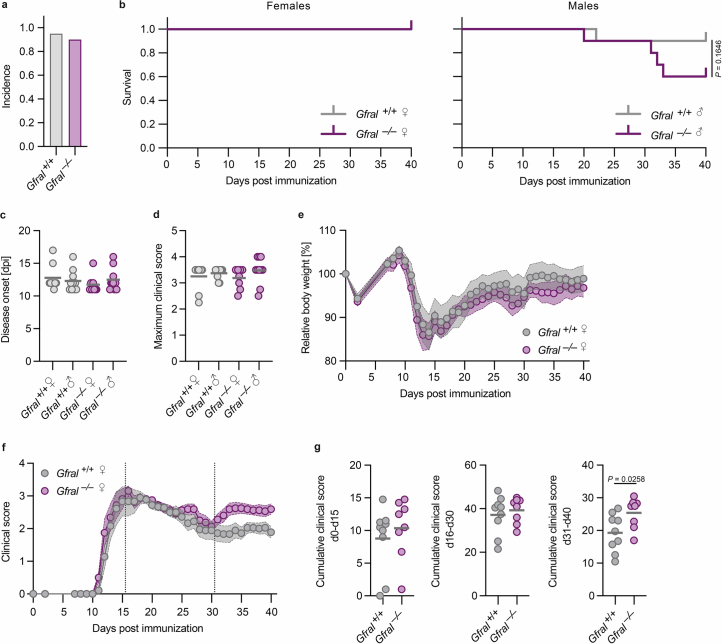
*Gfral* deficiency exacerbates neuroinflammation. EAE was induced in *Gfral*-proficient (*Gfral*^*+/+*^*, n* = 20) and -deficient (*Gfral*^*−/−*^*, n* = 20) mice. **a**, Incidence, **b**, survival, **c**, disease onset, and **d**, maximum clinical score in female (*Gfral*^*+/+*^*, n* = 9; *Gfral*^*−/−*^*, n* = 8) and male (*Gfral*^*+/+*^*, n* = 10; *Gfral*^*−/−*^*, n* = 10) littermates. **e**, Relative body weight loss and **f**, mean clinical disease score in female mice (*Gfral*^*+/+*^*, n* = 9; *Gfral*^*−/−*^*, n* = 8). **g**, Cumulative clinical score until acute phase (d0-d15), remission phase (d16-30) and relapse phase (d31-40) in female mice (*Gfral*^*+/+*^*, n* = 9; *Gfral*^*−/−*^*, n* = 8). Individual datapoints represent biological replicates. Data are shown as mean ± s.e.m. (**e**, **f**). For (**a**), a Fisher’s exact test was used. In (**b**) a Wilcoxon test was performed. For (**c**, **d**), a Kruskal-Wallis test with FDR correction was performed. In (**g**) a two-sided Mann-Whitney tests were performed. OE, overexpression. Source data

In mouse pregnancies with physiologically elevated GDF-15 levels (Extended Data Fig. 1b) and additional complex physiological changes, we did not observe downregulation of CD44 or LFA-1, but rather a slight increase, which was mitigated in *Gfral*-deficient dams (Extended Data Fig. 8h,i). Consistent with our observations in healthy mice receiving neuronal GDF-15 delivery, we found that loss of GFRAL abolished the protective effects in EAE (Fig. 5f–i). While *Gfral*-proficient mice showed negligible immune cell infiltration in the CNS, *Gfral*-deficient mice displayed substantial immune cell infiltration (Fig. 5j and Extended Data Fig. 8j), underscoring GFRAL as the key sensor that coordinates the effector mechanisms exerted on peripheral immunity during neuroinflammation. In line with these results, sensing of increasing levels of endogenously released GDF-15 during neuroinflammation was also critical to limit chronic tissue inflammation. Although we did not observe any difference in disease incidence (Extended Data Fig. 9a), male *Gfral*-deficient mice showed a trend toward decreased survival (Extended Data Fig. 9b), potentially because they failed to recover from acute inflammation. In female mice, we did not observe any impact on survival, disease onset or maximum clinical score with *Gfral* loss (Extended Data Fig. 9b–d). However, the failure to sense endogenously produced GDF-15 via GFRAL led to greater disease severity during the chronic disease phase (Extended Data Fig. 9e–g).

Given that we identified β-adrenergic signaling as a potential mediator of GDF-15 on peripheral T cells, we measured TH expression in the spleens of *Gfral*-deficient mice and controls after GDF-15 delivery. Notably, the loss of GFRAL resulted in significantly reduced splenic TH expression in response to GDF-15 (Fig. 5k), and a decrease in splenic norepinephrine levels (Fig. 5l). Furthermore, genetic deletion of *Gfral* restored LFA-1 expression on CNS-infiltrating CD4^+^ T cells after GDF-15 delivery (Fig. 5m).

**Extended Data Fig. 10:**
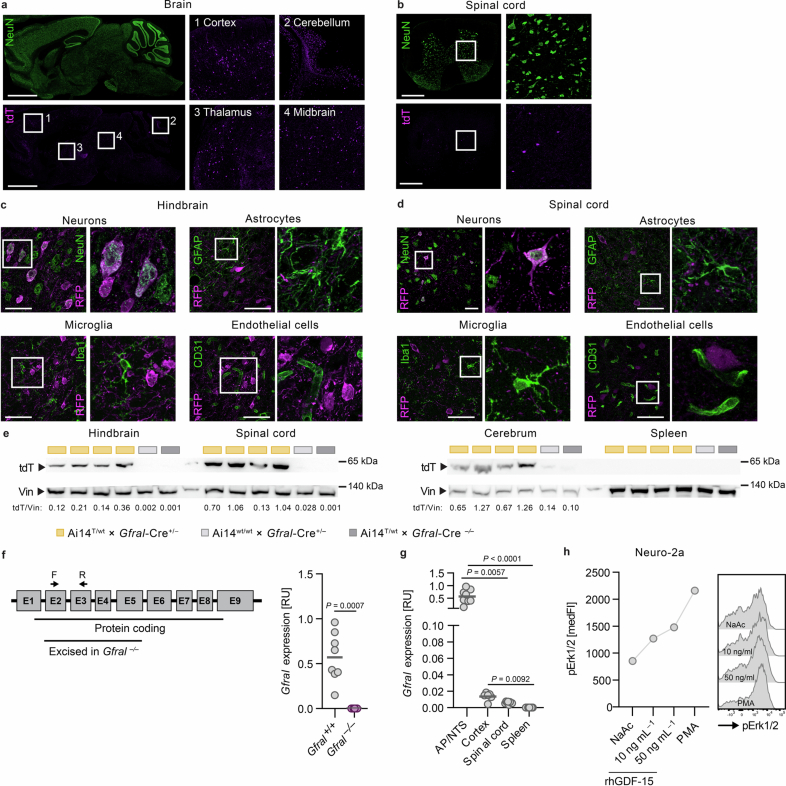
*Gfral* expression is limited to the CNS. **a**, Sagittal brain section of GFRAL-Cre × Ai14 reporter mice showing endogenous tdTomato (tdT) signal. Scale bar indicates 2 mm. **b**, Cervical spinal cord section of GFRAL-Cre × Ai14 reporter mice showing endogenous tdT. Scale bars, 500 µm. **c**, Z-stacks in hindbrain sections showing GFRAL-tdT^+^ cells counterstained with anti-RFP and for markers of neurons (NeuN^+^), astrocytes (GFAP^+^), microglia (Iba1^+^), and endothelial cells (CD31^+^). Scale bars, 50 µm. **d**, Z-stacks in cervical spinal cord sections showing GFRAL-tdT^+^ cells counterstained with anti-RFP and for markers of neurons, astrocytes, microglia, and endothelial cells. Scale bars, 50 µm. **e**, Immunoblot for RFP/tdTomato in tissue lysates from hindbrain, spinal cord, cerebrum, and spleen. *n* = 4 for experimental group Ai14^T/wt^ x *Gfral*-Cre^+/-^; *n* = 1 for each control. **f**, Scheme for oligonucleotide design targeting the mouse *Gfral* locus for RT-qPCR and validation of selected oligonucleotide pair in area postrema (AP)/nucleus tractus solitarius (NTS) tissue from *Gfral*^*+/+*^ (*n* = 8) and *Gfral*^*−/−*^ mice (*n* = 6). **g**, *Gfral* expression in healthy female and male C57BL/6J mice measured in AP/NTS (*n* = 8), cortex (*n* = 6), spinal cord (*n* = 8), and spleen (*n* = 7) by RT-qPCR. **h**, Neuro-2a cells were stimulated with increasing doses of recombinant human GDF-15 (rhGDF-15), 100 ng mL^*−*1^ PMA or NaAc for 30 minutes. Median fluorescence intensity (medFI) of pErk1/2 was determined by flow cytometry. Individual datapoints represent biological replicates. In (**a**-**d**) images from one representative animal are shown. In (**h**) data from one representative experiment is shown. For (**f**) a two-sided Mann-Whitney test was performed. In (**g**) a Kruskal-Wallis test with FDR correction was used. Source data

To visualize GFRAL-expressing cells with high sensitivity, we next generated GFRAL reporter mice by crossing GFRAL-Cre mice^23^ to Ai14 mice^51^. Consistent with earlier findings^23^, we observed tdTomato^+^ cells primarily in the area postrema and NTS region, with sparse labeling in other brain areas (Fig. 5n and Extended Data Fig. 10a). We also identified tdTomato labeling in the spinal cord (Extended Data Fig. 10b). Of note, tdTomato colocalized with the neuronal marker NeuN^+^, but not with astrocyte, microglia or endothelial cell markers (Extended Data Fig. 10c,d). We quantified tdTomato expression in the CNS and spleen using immunoblotting, confirming the absence of GFRAL expression in peripheral lymphoid organs (Extended Data Fig. 10e). Additionally, we validated the lack of *Gfral* expression in the spleen by quantifying mRNA expression (Extended Data Fig. 10f,g).

To exclude direct effects of GDF-15 on immune cells, we utilized phosphorylated Erk1/Erk2 (pErk1/pErk2) as a downstream signaling marker of GFRAL^21,22^. Both *Gfral*-proficient and *Gfral*-deficient leukocytes stimulated with recombinant GDF-15 showed no increase in pErk1/pErk2 levels (Fig. 5o), whereas this response was observed in Neuro-2a cells expressing both GFRAL and the co-receptor Ret (Extended Data Fig. 10h). In summary, our results indicate that GFRAL expression in the CNS is essential for GDF-15-induced protection from neuroinflammation via engagement of splenic β-adrenergic signaling.

### Selective activation of GFRAL^+^ neurons prevents neuroinflammation

**Fig. 6:**
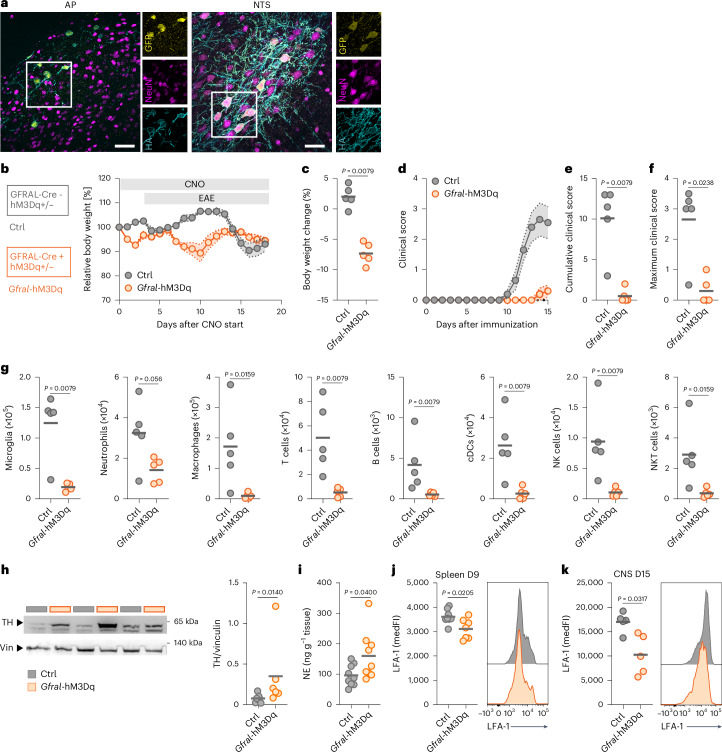
Chemogenetic activation of GFRAL^+^ neurons prevents neuroinflammation. **a**, Expression of hM3Dq in neurons of the area postrema (AP) and NTS in GFRAL-Cre × LSL-hM3Dq-DREADD (*Gfral*-hM3Dq) animals. Scale bars, 50 µm. **b**–**k**, Female and male mice expressing an activating DREADD in GFRAL^+^ cells (*Gfral*-hM3Dq) and Cre-negative littermates (Ctrl) received 4 µg ml^*−*1^ clozapine-*N*-oxide (CNO) in drinking water. Three days later, mice were immunized for EAE induction. **b**, Relative body weight. **c**, Body weight change after 48 h of CNO administration. **d**, Clinical score. **e**, Cumulative disease score. **f**, Maximum clinical score; *n* = 5 per group. **g**, Absolute number of microglia, macrophages, neutrophils, T cells, B cells, conventional dendritic cells (cDCs), natural killer (NK) cells and NK T cells in spinal cord quantified by flow cytometry on day 15 p.i.; *n* = 5 per group. **h**, Representative immunoblot for TH in the spleen on day 9 p.i. Expression was normalized to vinculin (Vin); *n* = 7 for Ctrl, *n* = 6 for *Gfral*-hM3Dq. **i**, Norepinephrine (NE) concentration in the spleen on day 9 p.i. normalized to tissue weight; *n* = 9 for Ctrl, *n* = 9 for *Gfral*-hM3Dq. **j**, MedFI of LFA-1 on CD4^+^ T cells in the spleen on day 9 p.i.; *n* = 8 for Ctrl, *n* = 7 for *Gfral*-hM3Dq. **k**, MedFI of LFA-1 on CD4^+^ T cells in the CNS during acute EAE (day 15 p.i.); *n* = 5 per group. Individual data points represent biological replicates. Data are shown as the mean ± s.e.m. (**b** and **d**). In **a**, data from one representative animal is shown. For **c**–**k**, two-sided Mann–Whitney tests were performed. Source data

Lastly, to test whether activation of GFRAL^+^ neurons is sufficient to replicate the effects of GDF-15 delivery, we used designer receptors exclusively activated by designer drugs (DREADD) technology^52^. We crossed GFRAL-Cre mice^23^ to LSL-hM3Dq-DREADD mice^53^, leading to expression of the mutant activating G-protein-coupled receptor hM3Dq specifically in GFRAL^+^ cells. We confirmed expression in area postrema and NTS neurons using an antibody directed against the hemagglutinin (HA)-tag, alongside visualization of the coexpressed mCitrine fluorochrome (Fig. 6a and Supplementary Data Fig. 9a,b). After administration of clozapine-*N*-oxide (CNO), which activates hM3Dq, only Cre^+^ mice showed 5–10% body weight loss (Fig. 6b,c). Chemogenetic activation of GFRAL^+^ neurons was sufficient to prevent clinical symptoms of neuroinflammation (Fig. 6d–f) and abolished immune cell infiltration into the spinal cord (Fig. 6g). Selective activation of GFRAL^+^ neurons also increased TH expression and norepinephrine accumulation in the spleen in the preclinical EAE phase (Fig. 6h,i). Additionally, LFA-1 expression was reduced in splenic CD4^+^ T cells (Fig. 6j) and CNS-invading CD4^+^ T cells (Fig. 6k). Thus, our data demonstrate that selective activation of GFRAL^+^ neurons induces β-adrenergic signaling in the spleen and effectively blocks neuroinflammation by preventing the infiltration of autoimmune T cells.

## Discussion

In this study, we investigated the role of GDF-15 in limiting neuroinflammation. Consistent with previous studies^17^, we identified that GDF-15 accumulates in human and mouse pregnancies as a consequence of changes in the fetal and maternal secretome. The observation that individuals with MS protected from relapses during pregnancy showed higher serum levels of GDF-15 prompted us to hypothesize that GDF-15 suppresses autoreactive T cells by engaging GFRAL on brainstem neurons. We identified GDF-15 as essential for restraining chronic CNS inflammation. Mice lacking GDF-15 experienced more severe EAE, failed to recover and exhibited a pro-inflammatory microglia and infiltrating myeloid cell phenotype. This finding is consistent with its tissue-protective properties during inflammation-induced cardiac failure^38^ and muscle injury^39^.

We further discovered that CNS-resident and infiltrating immune cells upregulate *Gdf15* expression during acute CNS inflammation. In addition to established molecular cues, such as the unfolded protein response^37^ and oxidative stress^54^, both hallmarks of MS^1^, T cell-derived cytokines such as IFNγ also induce *Gdf15* in neurons and glial cells. While previous studies suggested that IFNγ signaling raises systemic GDF-15 levels in infection models indirectly^55^, our data indicate that IFNγ directly induces GDF‑15 in neuroinflammation^2^.

Using gene therapy, we demonstrated that delivering GDF-15 to the CNS is sufficient to block immune cell infiltration and protect animals from EAE. Notably, this approach achieved plasma levels of GDF-15, comparable to those observed during late mouse pregnancy, when the suppression of autoimmune neuroinflammation is most pronounced^3,4^. Importantly, the protection from neuroinflammation could not be reproduced by caloric restriction, as pair-fed mice remained fully susceptible to EAE induction. In a complementary approach, administering recombinant native human GDF-15—originally developed to improve insulin sensitivity and treat obesity^21,22,44^—after EAE induction drastically alleviated neuroinflammation. Although clinical trials exploring GDF-15 for obesity treatment have been discouraging due to an insufficient reduction in body weight^56,57^, these studies have demonstrated the safety of administering GDF-15 analogs to humans. Our study suggests that the suppression of neuroinflammation by rhGDF-15 is uncoupled from body weight loss. Given the accessibility of brainstem GFRAL^+^ neurons via fenestrated capillaries^29^, clinically tested GDF-15 agonists may be repurposed to treat autoimmune diseases such as MS.

A previous report indicated that tumor-derived GDF-15 can impair T cell recruitment by limiting integrin-mediated adhesion to the vasculature, a process that can be reversed using a neutralizing GDF-15 antibody^24^. This approach has recently been successfully translated into clinical application for solid tumors^58^. However, the authors showed that this direct effect on immune cells is independent of GFRAL^24^, which up to now is the only known receptor for GDF-15^21,22^. Thus, we cannot exclude the possibility of GFRAL-independent immunomodulatory effects, as supported by a study showing that GDF-15 mediates anti-inflammatory effects in the liver in the presence of neutralizing anti-GFRAL antibodies, RET inhibitors or in *Gfral*-deficient mice^28^.

To resolve the discrepancy between GFRAL-dependent and GFRAL-independent effects, we combined a mutant GDF-15 lacking GFRAL-binding activity with studies in *Gfral*-deficient mice^50^, demonstrating that GDF-15-mediated protection from neuroinflammation is GFRAL dependent. Also, GDF-15, produced physiologically in response to neuroinflammation, acted as an endogenous brake to limit chronic tissue inflammation via activation of GFRAL^+^ neurons, since EAE was exacerbated in the late disease phase in *Gfral*-deficient mice. While the lack of GFRAL expression on immune cells has been postulated for many years, this conclusion mainly relied on mRNA expression analysis^24^. In contrast, our use of GFRAL reporter mice and functional assays on leukocytes now excludes direct GDF‑15–GFRAL engagement on immune cells. Instead, using DREADD technology^52,53^ to specifically activate GFRAL^+^ neurons, we showed that neuronal activation is sufficient to confer protection against neuroinflammation. This indicates that the purview of this small subpopulation of GFRAL^+^ brainstem neurons goes beyond metabolic adaptation in the context of thermoregulation, weight loss or energy expenditure^21,22,42,59^. Notably, our therapeutic approach was independent of corticosterone-induced immunosuppression via the hypothalamic–pituitary–adrenal axis^43^ and instead modulates immunity through the sympathetic nervous system, which activates β-adrenergic signaling in the spleen—a major reservoir of T cells that can access the CNS during neuroinflammation^60^.

Although earlier studies noted that GDF-15 stimulates triglyceride export in response to bacterial and viral infections^61^, it was not addressed whether central GFRAL^+^ neurons activation is required. While the projection landscape of GFRAL^+^ neurons has been mapped only within the CNS^23^, it is possible that they also project to peripheral anatomical regions such as the celiac plexus to regulate splenic immune responses, as previously shown for other brainstem neurons^62^. Further investigations are needed to decipher whether this regulation of peripheral immunity is mediated by CNS-intrinsic projections to different brain regions, or by projections to the peripheral nervous system that innervate lymphoid organs.

Mechanistically, we found that GDF-15 engagement of GFRAL downregulates the high-affinity conformation of the integrin LFA-1 and reduces T cell numbers in secondary lymphoid organs before the onset of EAE symptoms. This modulation is reversed by *Gfral* deletion and mimicked by chemogenetic activation of GFRAL^+^ neurons.

Although previous reports suggested that GDF-15 can directly modulate integrin activation on immune cells^24,38^, our findings indicate that central engagement of GFRAL in circumventricular organs by GDF-15 has a stronger influence on peripheral immunity. Furthermore, we showed that therapeutic GDF-15 delivery impairs peripheral T cell activation and proliferation. Based on these observations, we postulate that GDF-15-mediated impairment of T cell proliferation, activation and integrin expression in secondary lymphoid organs acts synergistically to protect from neuroinflammation. Inspired by previous studies on splenic sympathetic innervation affecting T cell exhaustion in cancer^49^, we demonstrated that activation of β-adrenergic signaling in the spleen can explain the modulation of autoimmune T cell responses. Ex vivo stimulation of T cells with norepinephrine and epinephrine, but also selective β_2_-adrenergic receptor agonists, decreased LFA-1 expression and activation of CD4^+^ T cells, whereas pharmacological inhibition or genetic deletion of ADRB2 abolished these effects.

**Fig. 7:**
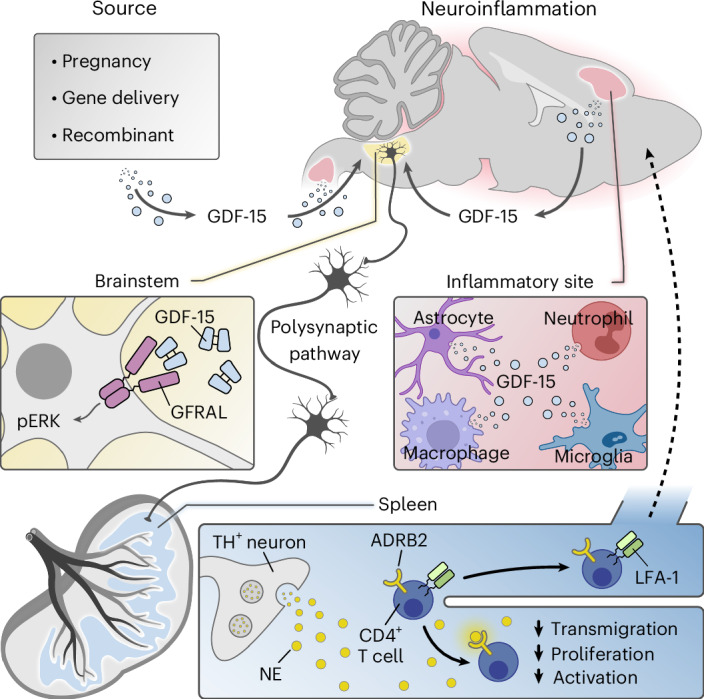
Working model of immunoception in neuroinflammation. High levels of GDF-15, released in response to pregnancy or in response to therapeutic delivery, or secreted from the inflamed tissue during neuroinflammation, are sensed by GFRAL-expressing neurons in the brainstem. Activation of a polysynaptic pathway downstream of GFRAL^+^ neurons stimulates β-adrenergic signaling in the spleen. As a consequence, TH^+^ neurons release NE and activate β_2_-adrenergic receptors (ADRB2) on CD4^+^ T cells, which dampens surface expression of the integrin LFA-1 and reduces activation and proliferation. This modulation of autoreactive T cells prevents transmigration to the CNS, thereby limiting neuroinflammation.

Based on our observations, we propose the following model (see graphical summary in Fig. 7): high levels of GDF-15, whether produced during pregnancy or in response to therapeutic delivery, or released within the inflamed neural tissue, enter the bloodstream and access central GFRAL^+^ neurons via fenestrated capillaries. Activation of these neurons triggers a polysynaptic cascade that leads to norepinephrine release from TH^+^ neurons into the spleen and possibly other secondary lymphoid organs. Engagement of β_2_-adrenoreceptors on CD4^+^ T cells results in downregulation of high-affinity LFA-1, and reduced T cell activation, proliferation and trafficking, ultimately protecting against neuroinflammation. This precise modulation of peripheral immunity by a small population of brainstem neurons exemplifies a fine-tuned evolutionary adaptation that controls aberrant autoimmune responses. It can, therefore, be considered a prime example of immunoception^11^, a term that was coined to describe the brain’s bidirectional monitoring and control of immunity. As such, our findings enhance our understanding of neuroimmune interactions and have broad implications for inflammatory diseases including MS.

A limitation of this study is that we could not detect the T cell-modulatory effects of therapeutic GDF-15 delivery in the context of mouse pregnancies. Although rAAV-mediated CNS expression effectively recalibrated T cell responses, high endogenous GDF-15 levels during pregnancy did not reduce LFA-1 expression or memory formation in CD4^+^ T cells. Consistently, genetic loss of *Gfral* in pregnant mice did not alter T cell responses, in contrast to the effects observed during CNS inflammation.

This context-specific difference may reflect a protective physiological adaptation. During pregnancy, excessive GDF-15 signaling can induce appetite loss and nausea, and rare genetic hyperactivation is linked to hyperemesis gravidarum^18^. To safeguard maternal nutrition, pregnancy may therefore attenuate GDF-15–GFRAL signaling. Supporting this possibility, membrane-bound matrix metalloproteinase 14 has been shown to mediate proteolytic cleavage of GFRAL and thereby reduce GDF-15 responsiveness in obesity^63^. A similar mechanism during pregnancy could limit peripheral immune recalibration despite elevated circulating GDF-15.

Future studies should explore how GFRAL expression and shedding are regulated during pregnancy and whether complementary immune checkpoints compensate to protect fetal development when GDF-15–GFRAL signaling is constrained. Additionally, the potential existence of an additional GDF-15 receptor remains unresolved, and its identification may help explain pregnancy complications associated with low GDF-15 levels^19^ despite the putative lack of GFRAL surface expression.

## Methods

### Participant cohorts

Indviduals with MS and healthy individuals were recruited through the MS outpatient clinic of the Department of Neurology, University Medical Center Hamburg-Eppendorf, and their blood samples were processed and stored by the Biobank of the Institute of Neuroimmunology and Multiple Sclerosis (Hamburger Patienteninformationssystem Multiple Sklerose, HAPIMS) approved by the local ethics committee (Hamburg Chamber of Commerce Act for the Health Professions, registration no. PV4405). Informed consent was obtained from all individuals. Blood samples were collected in polypropylene tubes (Sarstedt, S-Monovette, 7.5 ml; 01.1602) and processed within 2 h. The tubes were centrifuged at 1,840*g* for 10 min at 15 °C, and the supernatant was collected as serum. Pregnant women were recruited through the PRINCE (PRenatal IdeNtification of Children’s HEalth) study, which enrolled women of legal age experiencing a singleton pregnancy during their first trimester (gestational weeks 12–14). These participants were subsequently invited to attend two additional prenatal visits during the second trimester (gestational weeks 24–26) and third trimester (gestational weeks 34–36). Women with chronic infections (such as HIV and hepatitis B/C), known substance abuse issues or pregnancies resulting from assisted reproductive technologies were not eligible for participation. All participants provided informed consent, and the study protocol received approval from the ethics committee of the Hamburg Chamber of Physicians (license no. PV3694). Serum samples of pregnant women who experienced miscarriage or performed elective abortion were collected during routine blood sampling and processed according to standard laboratory methods at the Laboratory for Pediatric Rheumatology/Special Immunology at the University Hospital Wuerzburg (ethics protocol nos. 28/08 and 239/10). The study adhered to the principles outlined in the Declaration of Helsinki for medical research involving human participants. Blood was collected in polystyrene tubes and allowed to coagulate for 45 min. The samples were centrifuged at 1,500*g* for 20 min at 4 °C within 60 min of collection. All serum samples were aliquoted and stored at –80 °C until further analysis. The characteristics of the participants are shown in Supplementary Tables 1–4.

### Mice

All mice (C57BL/6J and BALB/c wild-type purchased from Charles River; *Gdf15*^*−/−*^^40^; *Gfral*^*−/−*^^50^, *Gfral*-Cre^23^ (purchased from the Jackson Laboratory, 036750), Ai14^51^, LSL-hM3Dq-DREADD^53^, *Gfral*-Cre × Ai14, *Gfral*-Cre × LSL-hM3Dq-DREADD and *Adrb2*^*−*/*−*^^64^ were kept under specific pathogen-free conditions in the central animal facility of the University Medical Center Hamburg-Eppendorf. Adult mice (6–20 weeks old) from both sexes were used, unless otherwise stated; mice were sex and age matched in all experiments. The mice were kept in a 12-h light–dark diurnal cycle, at 22°C ± 2 °C and 40–60% humidity, and given ad libitum access to standard chow (Altromin, 1328P) and water, unless otherwise stated. EAE mice additionally received DietGel Recovery (Ssniff; H007-72065). All animal care and experimental procedures were conducted in accordance with institutional guidelines and met the requirements of the German legal authorities. Ethical approvals were obtained from the State Authority of Hamburg, Germany (approval nos. 45/17, 007/22 and 108/24).

### EAE

Mice were immunized subcutaneously with 200 μg MOG_35–55_ peptide (peptides&elephants) in complete Freund’s adjuvant (BD Difco, 263.910) containing 2 mg ml^*−*^^1^ *Mycobacterium tuberculosis* (BD Difco, 231.141). Additionally, 300 ng of pertussis toxin (Merck Millipore, 516560-50UG) was injected intraperitoneally on the day of immunization and again 2 days later. Animals were scored daily for clinical signs using the following system: 0, no clinical deficits; 1, tail weakness; 2, hind limb paresis; 3, partial hind limb paralysis; 3.5, full hind limb paralysis; 4, full hind limb paralysis and fore limb paresis; 5, premorbid or dead. Animals reaching a clinical score ≥ 4 or not recovering from hind limb paralysis for more than 7 consecutive days were euthanized according to the regulations of the local Animal Welfare Act. For the analysis of chronic EAE (>day 15 after immunization), the clinical disease score for animals excluded from the analysis due to disease severity was carried forward as the mean of the group for statistical analysis. The cumulative clinical score represents the sum of the daily scores assigned to an animal over time. Whenever possible, mice were randomly assigned to treatment groups (rAAV, recombinant GDF-15) and mice from different experimental groups were housed together to minimize bias due to cage effects. Investigators were blinded to the genotype, the injected rAAV and treatment in the EAE experiments. For treatment with native human GDF-15 (Novo Nordisk), the protein was diluted in 0.9% NaCl, and mice received daily subcutaneous injections of 5 nmol per kg of body weight at a zeitgeber time of 2 ± 1. Control animals received a solution of 0.5 mM sodium acetate and 0.225% glycerol in 0.9% NaCl as a vehicle control. For DREADD activation, mice received 4 µg ml^*−*1^ CNO dihydrochloride (Cayman, 25780) in drinking water starting 3 days before EAE induction. Water was replaced every day, and consumption was monitored throughout the experiment.

### Viral injections

rAAVs were produced at the UKE vector facility using the PHP.eB capsid due to its high transduction efficacy in the CNS^65^. pUCmini-iCAP-PHP.eB was a gift from V. Gradinaru (Addgene plasmid, 103005). Viral genomes were determined by RT–qPCR using primers targeting the WPRE region. Mice were anesthetized with inhaled isoflurane (2–3%), and rAAVs were administered in 100 µl PBS by retrobulbar injection. Each mouse received a dose of 1 × 10^11^ viral genomes. Full transgene expression was observed 2 to 3 weeks after injection.

### Flow cytometric nucleus sorting

Isolation of nuclei from mouse spinal cords was performed as previously described^66^. Briefly, mice were euthanized with CO_2_ and perfused with cold PBS. Spinal cords were dissected and stored at –80 °C. The tissue was mechanically dissociated with a scalpel, added to 2 ml of EZ buffer (Sigma-Aldrich, NUC101) and dissociated using a glass douncer (Sigma-Aldrich, D9063). After a 5-min incubation on ice, the nuclei were pelleted by centrifugation (500*g*, 5 min, 4 °C) and the pellet was washed in 2 ml of EZ buffer, followed by two washing steps in nuclei incubation buffer (340 mM sucrose, 2 mM MgCl_2_, 25 mM KCl, 65 mM glycerophosphate, 5% glycerol, 1 mM EDTA, 1% bovine serum albumin or BSA). Nuclei were filtered through a 30-μm filter, followed by staining with AF647-labeled NeuN antibody and 0.25 µg ml^*−*1^ propidium iodide (BioLegend, 421301) as a DNA counterstain. NeuN^+^ and NeuN^*−*^ single nuclei were sorted using a BD FACSAria III cell sorter (BD Biosciences) with a 70-µm nozzle. RNA for real-time PCR was isolated as described below.

### Flow cytometric cell sorting

For isolation of astrocytes and immune cells from spinal cord tissue, we adapted a protocol by Scheyltjens et al.^67^, incorporating the transcriptional inhibitor actinomycin D (ActD) throughout the workflow. Spinal cord tissue from EAE animals and healthy controls was collected in RPMI-1640 medium (PAN Biotech, P04-18500) supplemented with 25 mM HEPES (Gibco, 15630056) and 30 µM ActD (Cell Signaling, 15021S) after transcardial PBS perfusion. Tissue was dissociated into single-cell suspensions in 1 mg ml^*−*1^ collagenase A (Roche, 11088793001) and 200 IU ml^*−*1^ DNase I (Merck Millipore, 260913) using the gentleMACS Octo Dissociator (Miltenyi Biotec, program: Multi_F). The dissociated tissue was applied to a 70-µm cell strainer, and the filter was rinsed three times with RPMI-1640 supplemented with 25 mM HEPES and 3 µM ActD. Dissociated tissue was collected after centrifugation at 500*g* for 5 min at 4 °C, and immune and glial cells were enriched using a discontinuous density gradient with Percoll PLUS (GE Healthcare, 17-5445-01). Isotonic Percoll solutions were prepared with HBSS and supplemented with 3 µM ActD. After centrifugation at 1,350*g* and 4 °C for 30 min, cells were collected from the interphase between the 30% Percoll and 70% Percoll layer. Cells were washed in FACS buffer (PBS, 1 mM EDTA, 1% BSA (Miltenyi Biotec, 130-091-376), 10 mM HEPES) at 650*g* and 4 °C for 10 min. Nonspecific Fc receptor-mediated antibody binding was blocked by pre-incubation with TruStain FcX anti-mouse CD16/CD32 antibody (BioLegend, 101320) for 10 min at 4 °C before staining with surface antibodies in FACS buffer for 20 min at 4 °C. All antibodies used in this study are listed in Supplementary Table 7. Cells were washed and resuspended in FACS buffer supplemented with 0.4 U µl^*−*1^ RiboLock RNase Inhibitor (Thermo Fisher Scientific, EO0382) and 2.5 µM Helix NP Green (BioLegend, 425303) to exclude dead cells. Up to 70,000 cells were sorted into DNA LoBind tubes (Eppendorf, 0030108051) filled with 700 µl RLT buffer supplemented with 40 mM dithiothreitol (Roche, 10197777001) using a BD FACSAria III cell sorter (BD Biosciences) equipped with a 100-µm nozzle. RNA for real-time PCR was processed as described below.

### Real-time PCR

RNA was extracted using the RNeasy Mini Kit (Qiagen) with DNase I treatment and subsequently reverse-transcribed to complementary DNA using the RevertAid H Minus First Strand cDNA Synthesis Kit (Thermo Fisher Scientific) according to the manufacturer’s instructions. Gene expression was analyzed by the QuantStudio Flex 6 Real-Time PCR System (Applied Biosystems) using TaqMan Gene Expression Assays (Thermo Fisher Scientific) for *Gdf15* (Mm00442228_m1), *Tbp* (Mm01277042), *Adrb1* (Mm00431701_s1), *Adrb2* (Mm02524224_s1) and *Adrb3* (Mm00442669_m1) or PowerUp SYBR Green Master Mix (Applied Biosystems) using custom-made oligonucleotides for *Gfral* and *Tbp* (Supplementary Table 8). Primer design was performed using a built-in algorithm in Benchling. All analyses were performed in technical duplicates or triplicates. We calculated gene expression as 2^*−*ΔCt^ relative to *Tbp* as the endogenous control.

### Cell lines

Neuro-2a cells and HEK 293T cells were obtained from DSMZ (ACC 148) and passaged in DMEM high glucose (Gibco, 61965059) supplemented with 10% fetal bovine serum (Pan Biotech, P30-3306) and 1% penicillin–streptomycin (Gibco, 15070063). SIM-A9 microglial cells were obtained from Biocat (T0247-GVO-ABM) and passaged in DMEM:F12 (Pan Biotech, P04-41250) supplemented with 10% heat-inactivated fetal bovine serum (Sigma-Aldrich, F7524), 5% heat-inactivated horse serum (Pan Biotech, P30-0702) and 1% penicillin–streptomycin. All cell lines were maintained at 37 °C and 5% CO_2_, and regularly checked for mycoplasma contamination using the VenorGeM Advance kit (Minerva Biolabs, 11-7024) according to the manufacturer’s instructions. To generate stable cell lines expressing the *Gdf15*-mScarlet reporter construct, Neuro-2a and SIM-A9 microglial cells were transduced with lentiviral particles in the presence of 8 µg ml^*−*1^ polybrene (Sigma-Aldrich, H9268), and mScarlet^+^ cells were isolated using a BD FACSAria III cell sorter. Samples were supplemented with 10 µM DAPI (BioLegend, 422801) before acquisition to exclude dead cells.

### Primary neuronal and astrocytic cultures

For primary cortical cultures, pregnant C57BL/6J mice were euthanized at gestational day 15.5. The cortex was isolated and dissociated, and cells were plated at a density of 6 × 10^4^ per cm^2^ on poly-D-lysine-coated wells (5 µM, Sigma-Aldrich, P6407). For immunocytochemistry, primary cortical neurons and astrocytes were cultivated on poly-D-lysine-coated 12-mm diameter coverslips. Unless otherwise stated, cells were maintained in Neurobasal Plus medium (supplemented with B-27 Plus, penicillin, streptomycin, and L-glutamine; Gibco, A3582901) at 37 °C and 5% CO_2_, and a relative humidity of 98%. Half-medium exchanges were performed every 3 to 4 days. Throughout this study, cultures after 14 to 18 days in vitro were used for experiments.

### Primary T cell cultures

For primary T cell cultures, spleens and lymph nodes from C57BL/6J mice were collected and processed into a single-cell suspension using 70-µm cell strainers. Red blood cells in splenocytes were lysed by incubation in erythrocyte lysis buffer (10 mM potassium bicarbonate, 0.15 M ammoniochloride, 0.1 mM Na_2_EDTA in double-distilled water; pH 7.4) for 2 min. Spleen and lymph node cells were pooled, and T cells were enriched using the MojoSort Mouse CD3 or CD4 T cell Isolation Kit (BioLegend, 480024 or 480006, respectively) according to the manufacturer’s instructions. Flat-bottom cell culture plates were coated with 0.5 µg ml^*−*1^ anti-CD3 (BioLegend, 100238), and cells were seeded in T cell medium (RPMI-1640, 10% heat-inactivated fetal bovine serum, 1% penicillin–streptomycin, 10 mM HEPES, 50 µM 2-mercaptoethanol (Gibco, 31350010), 1% GlutaMAX (Gibco, 35050061), 1 mM sodium pyruvate (Gibco, 11360070) and 1% non-essential amino acids (Gibco, 11140050)) supplemented with 0.5 µg ml^*−*1^ anti-CD28 (BioLegend, 102116) and 25 IU ml^*−*1^ recombinant mouse IL-2 (PeproTech, 212-12). For proliferation assays, T cells were stained with 5 µM CellTrace CFSE (Invitrogen, C34554) before seeding according to the manufacturer’s instructions.

### Compounds and chemicals

Compounds were added to primary cells or cell lines at the indicated time points specified in the respective figure legends. Unless otherwise stated, cells were stimulated every 24 h. The following concentrations were used: 20 or 100 ng ml^*−*1^ recombinant mouse IFNγ (PeproTech, 315-05), 20 ng ml^*−*1^ recombinant mouse GM-CSF (PeproTech, 315-03), 1 µg ml^*−*1^ tunicamycin (Sigma-Aldrich, T7765), 250 µg ml^*−*1^ 4-ocytl itaconate (Hycultec, HY-112675), 10 µM norepinephrine (Cayman, Cay16673), 10 µM epinephrine (Sigma-Aldrich, E4250), 10 µM xamoterol (Cayman, Cay24267), 10 µM indacaterol (Cayman, Cay20070), 10 µM CGP 20712A (Cayman, Cay40765-1), 10 µM ICI 118551 (Hycultec, HY-13951), 100 ng ml^*−*1^ lipopolysaccharide (Sigma-Aldrich, L4391) and 10 ng ml^*−*1^ or 50 ng ml^*−*1^ native human GDF-15 (Novo Nordisk).

### Immunocytochemistry, immunohistochemistry and imaging

Cells were fixed with 4% paraformaldehyde and incubated in 5% normal donkey serum (NDS) containing 0.1% Triton X-100. Immunolabeling was performed in 2% NDS and 1% BSA in 0.1% Triton X-100. All antibodies used in this study and their dilutions are provided in Supplementary Table 7. Images were acquired using a confocal LSM 900 laser scanning confocal microscope (Zeiss). We quantified the endogenous intracellular mScarlet signal in neurons (NeuN^+^) and astrocytes (GFAP^+^) using ImageJ 1.54i. For each biological replicate, we calculated the mean from three regions of interest per coverslip. Mouse spinal cord, brain and spleen tissue were obtained and processed as described previously^68^. Briefly, cryosections from perfused and paraformaldehyde-fixed mid-cervical spinal cords, brain or spleen were incubated in 5% NDS containing 0.1% Triton X-100 and were subsequently stained with antibodies listed in Supplementary Table 7. For antibodies of mouse origin, sections were additionally incubated with AffiniPure Fab fragment donkey anti-mouse IgG (H+L; Jackson ImmunoResearch) for 60 min at room temperature. Images were acquired using an LSM 900 laser scanning confocal microscope (Zeiss) and analyzed using ImageJ 1.54i. For the quantification of Iba1 and GFAP, a mask for the gray matter or white matter was manually applied, and the MFI was calculated for each region of interest. For the quantification of absolute cell counts (CD45, CD68, CD3), all positive cells per spinal cord section were counted. During quantification in Fiji, the investigators were blinded to the experimental group.

### Immune cell isolation for flow cytometry

iLNs and spleen samples were homogenized through a 70-µm cell strainer and washed with PBS (500*g*, 5 min, 4 °C). Red blood cells were lysed as described above. Brain and spinal cord tissue were collected after transcardial PBS perfusion and dissociated into single-cell suspensions in 1 mg ml^*−*1^ collagenase A and 0.1 mg ml^*−*1^ DNase I using the gentleMACS Octo Dissociator (program: Multi_F). The dissociated tissue was applied to a 70-µm cell strainer, and immune and glial cells were enriched using a discontinuous density gradient (GE Healthcare, GE17-0891-01). Cells were collected from the interphase as described above. Nonspecific Fc receptor-mediated antibody binding was blocked by pre-incubation with TruStain FcX anti-mouse CD16/CD32 antibody before staining of surface antibodies in Brilliant Stain Buffer (BD Biosciences) for 30 min at 4 °C. For staining of intranuclear proteins cells were fixed in 1× Fixation/Permeabilization working solution for 45 min at 4 °C, followed by incubation with antibodies targeting Nur77 or Ki67 in 1× Permeabilization buffer for 45 min at 4 °C (Invitrogen, 00-5523). All antibodies used in this study are listed in Supplementary Table 7. We excluded dead cells from the analysis by staining with Zombie Aqua, Green, Yellow and NIR Fixable Viability Stains (BioLegend, 423101, 423112, 423104, 423106) or 0.8 µM Alexa Fluor 750 NHS (Invitrogen, A20011). For the determination of absolute cell numbers, CD45^hi^ leukocytes and CD45^int^ microglia were quantified using Precision Count Beads (BioLegend, 424902). Data were obtained using a BD Symphony A3 flow cytometer (BD Biosciences) and analyzed using FlowJo version 10.9 (BD Biosciences). During the analysis in FlowJo, the investigators were blinded to the experimental group.

### RNA sequencing and analysis

RNA was isolated from CD4^+^ enriched T cells using the RNeasy Mini Kit (Qiagen) with DNase I treatment. RNA-sequencing libraries were prepared using the TruSeq stranded mRNA Library Prep Kit (Illumina) according to the manufacturer’s instructions. Libraries were pooled and sequenced on a NovaSeq 6000 sequencer (Illumina), generating 150-base-pair, paired-end reads with poly-A enrichment. The reads were aligned to the Ensembl mouse reference genome (GRCh39) using STAR v2.7.9a with default parameters for paired-end reads. Gene assignments were quantified with featureCounts v1.5.1. Differential expression analysis used DESeq2 v1.40.2 with an FDR-adjusted significance threshold of *P* < 0.05. Differentially expressed genes were annotated using biomaRt v2.56.1. Sample similarity was assessed through principal component analysis utilizing the top 500 genes with the highest variance of log_2_-normalized counts. Gene expression heat maps and visualizations were generated with ggplot2 v3.4.3 for comprehensive data representation.

### Immunoblot

Tissue from EAE and healthy mice were homogenized in radioimmunoprecipitation buffer (50 mM Tris, 150 mM NaCl, 0.5 mM EDTA, 10% SDS, 1% NP-40, 10% sodium deoxycholate, plus protease and phosphate inhibitor cocktails (PhosSTOP Merck, 4906845001 and cOmplete, Sigma-Aldrich, 11836170001)) using a Qiagen TissueLyser LT with the setting 1/50s for 1 min and incubated at 4 °C for 30 min on a rotating wheel. The lysates were centrifuged for 10 min at 16,000*g* to remove the cell debris. Protein concentrations were determined by BCA assay (Pierce BCA Protein Assay Kit, Thermo Fisher Scientific, 23228 and 23224) according to the manufacturer’s protocol. Samples were prepared using Western-Ready Protein Sample Loading Buffer (5×; BioLegend, 426311) and boiled at 95 °C for 10 min. A total of 10 µg of protein was loaded onto 8–12% gradient or 10% non-gradient SDS–PAGE (NuPAGE, Thermo Fisher Scientific, NW04125BOX, NW00105BOX) followed by wet transfer to polyvinylidene fluoride membranes. Blocking was performed using 5% milk powder in 1× TBS-T for 1 h at room temperature. Membranes were incubated with primary antibodies overnight at 4 °C. Horseradish peroxidase-coupled secondary antibodies were applied for 1 h at room temperature, and chemiluminescence was visualized using WesternSure PREMIUM Chemiluminescent Substrate (LI-COR, 926-95000) according to the manufacturer’s protocol. All antibodies used are listed in Supplementary Table 7.

### Statistics and reproducibility

The statistical analyses applied during the bioinformatics analysis are detailed in the respective sections. Unless otherwise stated, the data are presented as means ± s.e.m. or the mean alone if individual data points for biological replicates are displayed. Data distribution was assumed to be normal, but this was not formally tested. All statistical analyses were performed using Prism 9.5.1 (GraphPad) as indicated in the figure legends. Corrections for multiple testing were controlled by the FDR (Benjamini and Hochberg). A *P* value of ≤0.05 was considered significant.

To determine sample sizes, we performed a power analysis using G*Power 3.1 with the following parameters based on previous studies^66,68^ and existing preliminary data: two-sided Wilcoxon–Mann–Whitney test; type I error, 0.05; type II error, 0.2; effect size based on Cohen, 1 to 2.5 (varies between experimental setups).

Whenever immunohistochemical stainings of reporter mice are shown, we analyzed sections from at least three individual animals and selected representative images.

### Reporting summary

Further information on research design is available in the Nature Portfolio Reporting Summary linked to this article.

## Online content

Any methods, additional references, Nature Portfolio reporting summaries, source data, extended data, supplementary information, acknowledgements, peer review information; details of author contributions and competing interests; and statements of data and code availability are available at 10.1038/s41590-025-02406-1.

## Supplementary information

Supplementary Information Supplementary Methods, Figs. 1–9 and Tables 1–4

Reporting Summary

Supplementary Tables 5–9 Supplementary Table 5 | Regulated metabolites in plasma from rAAV-injected animals. *P* values were adjusted for multiple testing using FDR correction. Only metabolites with a *P*_adjust_ < 0.05 and a log_2_-fold change ≥ 0.5 are displayed. Supplementary Table 6 | Regulated metabolites in plasma from rAAV-injected animals 9 days after EAE induction. *P* values were adjusted for multiple testing using FDR correction. Only metabolites with a *P*_adjust_ < 0.05 and a log_2_-fold change ≥ 0.5 are displayed. Supplementary Table 7 | Antibodies used in this study. Supplementary Table 8 | Oligonucleotides used in this study. Supplementary Table 9 | Raw data of plasma metabolomics.

Supplementary Data 1 Statistical source data for Supplementary Figs. 2 and 4–8.

## Source data

Source Data Fig. 1 Statistical source data.

Source Data Fig. 2 Statistical source data.

Source Data Fig. 3 Statistical source data.

Source Data Fig. 4 Statistical source data.

Source Data Fig. 5 Statistical source data.

Source Data Fig. 6 Statistical source data.

Source Data Extended Data Fig. 1 Statistical source data.

Source Data Extended Data Fig. 2 Statistical source data.

Source Data Extended Data Fig. 3 Statistical source data.

Source Data Extended Data Fig. 4 Statistical source data.

Source Data Extended Data Fig. 5 Statistical source data.

Source Data Extended Data Fig. 6 Statistical source data.

Source Data Extended Data Fig. 7 Statistical source data.

Source Data Extended Data Fig. 8 Statistical source data.

Source Data Extended Data Fig. 9 Statistical source data.

Source Data Extended Data Fig. 10 Statistical source data.

Source Data Figs. 4–6 and Extended Data Figs. 6 and 10 Unprocessed immunoblot images.

## Data Availability

Sequencing data generated for this study have been deposited in the Gene Expression Omnibus under accession code GSE288193. All other data are available in the main text or the Supplementary Information. Source data are provided with this paper.

## Code availability

The code used for the study can be obtained from the corresponding author upon reasonable request.
