# Supplementary material for: A GDF-15–GFRAL axis controls autoimmune T cell responses during neuroinflammation
Source: Nat Immunol. 2026 Jan 15;27(3):503–15. doi: 10.1038/s41590-025-02406-1 (PMC12956584; doi:10.1038/s41590-025-02406-1)
Supplement: Supplementary file 1 — Supplementary Methods, Figs. 1–9 and Tables 1–4 [file 41590_2025_2406_MOESM1_ESM.pdf]

nature immunology

Supplementary information https://doi.org/10.1038/s41590-025-02406-1

A GDF-15–GFRAL axis controls autoimmune T cell responses during neuroinflammation

In the format provided by the authors and unedited

SUPPLEMENTARY INFORMATION

SUPPLEMENTARY METHODS

Syngeneic and semi-allogeneic pregnancy. Female mice were primed with housing material from fertile C57BL/6J or BALB/c males for two days and then mated for three consecutive nights. Successfully mated females were identified by the presence of a vaginal plug, separated, and weighted after ten days to confirm pregnancy. The day of plug was considered gestational day (GD) 0.5. Experiments were performed on GD17.5 ± 1.

GDF-15 enzyme-linked immunosorbent assay. For the generation of tissue lysates, snap-frozen spinal cord or cortex tissue was immersed in 500 µL extraction buffer per 100 mg of tissue weight. The extraction buffer consists of 100 mM Tris-HCl, 150 mM $NaCl_2$, 1 mM EGTA, 1 mM EDTA, 1% Triton-X100 and 0.5% sodium deoxycholate. Tissues were lysed at 50 Hz for 1 min using a Tissue Lyser LT (Qiagen). Mouse plasma and serum from pregnant women were collected according to standard procedures and stored at –80 °C. A commercial ELISA kit for murine GDF-15 (R&D systems) was modified using clone 01G06 as GDF-15 capture antibody (Evitria) to determine the GDF-15 levels in mouse plasma and tissue. For the analysis of GDF-15 in serum from healthy PRINCE pregnancies and women who had miscarriage or abortion, a commercial ELISA kit targeting human GDF-15 (R&D systems) was modified by replacing the detection antibody with an in-house generated GDF-15 antibody (patent: EP3122775A1) as previously described[66]. For the analysis of GDF-15 in serum from pregnant MS patients a commercial ELISA kit targeting human GDF-15 (R&D systems) was used.

Norepinephrine enzyme-linked immunosorbent assay. For the generation of tissue lysates, spleen tissue was collected in liquid nitrogen. Within less than four hours, the tissue was immersed in 100 µL stabilizing buffer per 10 mg of tissue, consisting of 1/20 of stabilizer in distilled H2O. The tissue was further lysed in the stabilizing buffer, using a Tissue Lyser LT (Qiagen) at 50 Hz for 2 min at 4°C, and centrifuged at 16.000 × g for 5 min at 4°C. Supernatant was collected and stored at -20°C. Mouse plasma was collected and supplemented with stabilizer (20% of the plasma volume), and stored at -20°C. To determine the norepinephrine levels in mouse spleen tissue lysates and plasma, a commercial ELISA kit for norepinephrine (DLD, Noradrenaline High Sensitive ELISA, EA633/96) was used, and samples were processed according to the manufacturer's instructions.

Metabolic cages. Indirect calorimetry was conducted using Promethion metabolic cages (Sable Systems). Mice were acclimated for ≥ 2 days at 22 °C. Oxygen consumption (VO_2), carbon dioxide production (VCO_2) and food intake were continuously monitored. Data analysis was performed using Sable Systems' Macro interpreter software. Respiratory exchange ratio (RER) was calculated as VCO_2/VO_2.

Paired feeding. Mice were housed in groups of two to three per cage based on the rAAV to minimize stress induced by single housing. From day 7 post-AAV injection onwards the amount of consumed food was monitored, and the delta (g) consumed by the GDF-15 OE group was determined. GFP OE mice received either food *ad libitum* diet, or a restricted amount of food equivalent to the mean consumption of the GDF-15 OE group during the previous 24 hours interval.

Vector construction. All primers used in this study are provided in **Supplementary Table 8**. Primer design was performed using a built-in algorithm in Benchling. The promoter sequence of mouse *Gdf15* was amplified from Neuro-2a cell lysates. The PCR amplification product was digested with the restriction enzymes PacI and BshTI and ligated into a customized lentiviral backbone with an mScarlet cassette downstream of the promoter sequence to assess GDF-15 promoter activity by fluorochrome expression. The sequence of full-length murine *Gdf15* was amplified from Neuro-2a cDNA. The PCR product was digested with Pfl23II and SacI and ligated into a customized pAAV backbone with a human synapsin 1 (hSyn1) promoter derived from pAAV-hSyn1-mTurquoise2, two nuclear localization signals (NLS; N- and C-terminal), eGFP, and a P2A cleavage peptide. pAAV-hSyn1-mTurquoise2 was a gift from Viviana Gradinaru (Addgene plasmid #99125; http://n2t.net/addgene:99125; RRID:Addgene_99125). For the control plasmid, a stop codon was inserted after the P2A cleavage peptide sequence. A recombinant PCR was used to replace GTT, coding for valine, by AGG, coding for arginine, at amino acid position 90 of the mature peptide (position 278 of the full-length protein). All final products were confirmed with Sanger sequencing.

Lentivirus production. To produce VSV-G-pseudotyped lentiviruses, HEK293T cells (ACC 635; DSMZ, Leibniz, Germany) were seeded at 6×10^4 per cm^2 one day prior to transfection in DMEM with glutamine and high glucose (Thermo Fisher Scientific). Helper plasmids pMDLg/pRRE, pRSV-Rev and pMD2.G were a kind gift from Didier Trono (Addgene #12251; Addgene #12253; Addgene #12259). For a 10 cm cell culture plate we used 15 µg transfer plasmid, 10 µg

pMDLg/pRRE, 5 µg pRSV-Rev, and 2 µg pMD2.G. Plasmids were mixed in 1× HEPES-buffered saline (HBS) and a 125 mM $CaCl_2$ solution and applied to the HEK293T cells in the presence of 25 µM chloroquine diphosphate. After 12 hours, the medium was changed, and the supernatant was collected 36 hours post-transfection, filtered through a 0.45 µm PES filter, and immediately snap-frozen and stored at –80 °C. The lentiviral particles were concentrated using the Lenti-X Concentrator (Takara Bio) according to the manufacturer's instructions. Primary cortical cultures were transduced at days in vitro (DIV) 7.

Metabolomics. Metabolomic profiling of plasma samples was carried out using the MxP® Quant 500 XL Kit (BIOCRATES Life Sciences AG), enabling the identification and quantification of up to 1,019 metabolites using liquid chromatography–tandem mass spectrometry (LC-MS/MS) and flow injection analysis (FIA). Plasma samples were processed in accordance with the manufacturer's instructions. Briefly, 10 µL of plasma, calibration standards, and control samples were transferred onto two filter plates preloaded with internal standards for calibration. One filter plate was allocated for LC-MS/MS measurements and the first FIA set, while the second plate was used exclusively for a second FIA set. Filter plates were dried under a nitrogen stream using a positive pressure manifold (Waters Cooperation). For LC-MS/MS analysis, the first plate was incubated with the derivatization reagent phenyl isocyanate (Merck KGaA) for 60 minutes, followed by a second drying step. After drying, metabolites were extracted using 5 mmol L^{-1} ammonium acetate in methanol. The resulting eluates were diluted either in LC-MS-grade water (for LC-MS/MS analysis) or FIA solvent (for FIA measurements). All analyses were conducted using an ACQUITY UPLC I-Class system coupled to a Xevo TQ Absolute mass spectrometer (Waters Cooperation). Reversed-phase chromatographic separation was carried out on a C18 LC column (BIOCRATES) using an eluent system of 0.2% formic acid in water and 0.2% formic acid in acetonitrile. FIA solvent consisted of methanol modified according to the manufacturer's instructions. All measurements were performed in both positive and negative ionization mode. Data analysis was executed using the WebIDQ cloud-based software package (BIOCRATES, version 2024). Metabolite concentrations were quantified using either a seven-point calibration curve or one-point calibration with internal standard normalization. The raw data is accessible in **Supplementary Table 9**. Only metabolites with a false discovery rate-adjusted significance threshold of $P < 0.05$ and a log2-fold change ≥ 0.5 were included into the analysis.

SUPPLEMENTARY DATA FIGURES

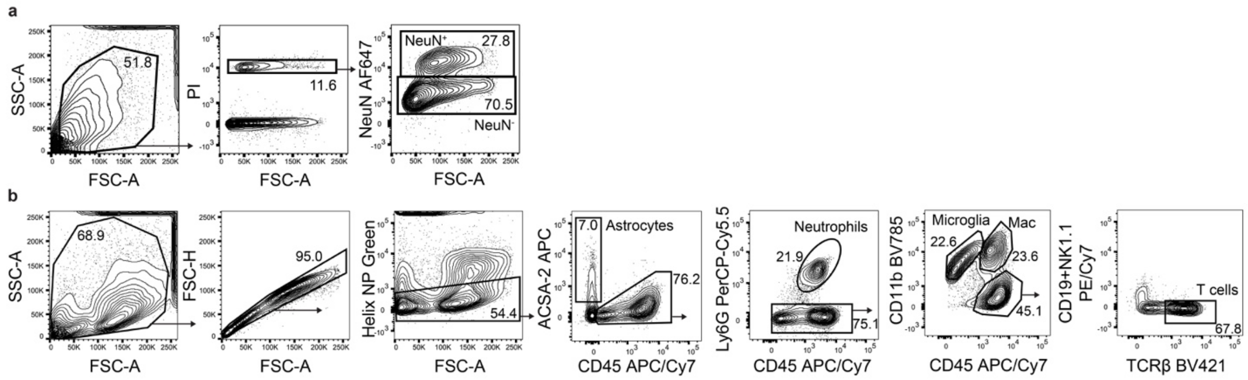

Supplementary Data Fig. 1 | Sorting of nuclei and cells from spinal cord tissue. a, Representative gating strategy to isolate NeuN$^+$ and NeuN$^-$ nuclei from spinal cords by flow cytometry. **b**, Representative gating strategy to isolate astrocytes, microglia and central nervous system (CNS)-infiltrating immune cells from spinal cord tissue by flow cytometry.

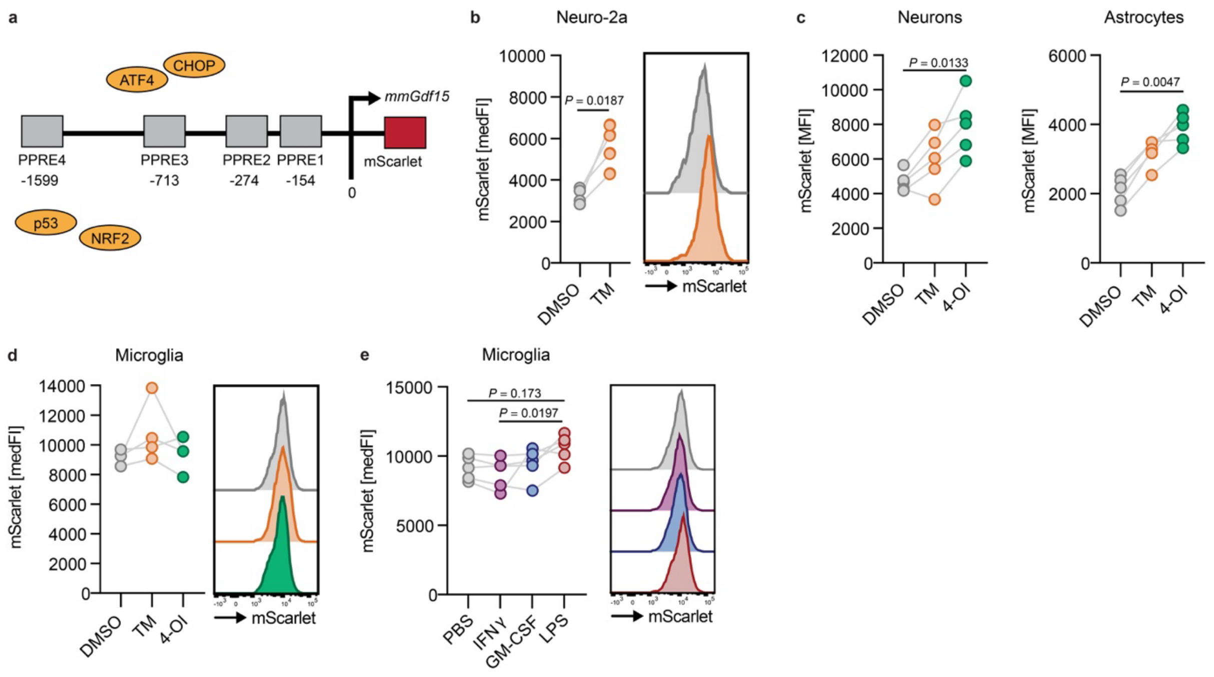

Supplementary Data Fig. 2 | Genetic construct reports *Gdf15* promoter activity in various cell types. a, Scheme of *Gdf15* promoter region with putative PPARγ response elements (PPRE) adapted from Lu et al.[36]. Activation of the promoter region induces expression of the orange fluorochrome mScarlet. **b**, Neuro-2a cells were transduced with the P_{Gdf15}-mScarlet reporter lentivirus, and stably expressing cells were isolated by flow cytometry. Cells were stimulated with 1 µg mL^{-1} tunicamycin (TM) for 48 hours prior to quantification of the median fluorescence intensity (medFI) mScarlet signal by flow cytometry. Dead cells were excluded by addition of 10 µM DAPI prior to acquisition; *n* = 4. **c**, Primary cortical neuron-astrocyte co-cultures were transduced with the P_{Gdf15}-mScarlet reporter lentivirus and stimulated with 1 µg mL^{-1} TM or 250 µg mL^{-1} 4-octylitaconate (4-OI) for 24 hours. The mean fluorescence intensity (MFI) was quantified separately in neurons ($NeuN^+$) and astrocytes ($GFAP^+$) by immunocytochemistry; *n* = 5. **d, e**, mScarlet expression was quantified as median fluorescence intensity (medFI) in stably transduced SIM-A9 microglia cells by flow cytometry. Dead cells were excluded by addition of 10 µM DAPI prior to acquisition. **d**, Microglia cells were stimulated with 1 µg mL^{-1} TM or 250 µg mL^{-1} 4-OI for 48 hours; *n* = 4 independent experiments. **e**, Microglia cells were treated with 20 ng mL^{-1} IFNγ, 20 ng mL^{-1} GM-CSF or 100 ng mL^{-1} LPS for 48 hours; *n* = 5. Individual datapoints represent independent experiments (**b**, **d**, **e**) or biological replicates (**c**). In (**b**) a two-sided paired *t*-test was used. In (**c-e**) a Friedman test with FDR correction was performed. RU = Relative units.

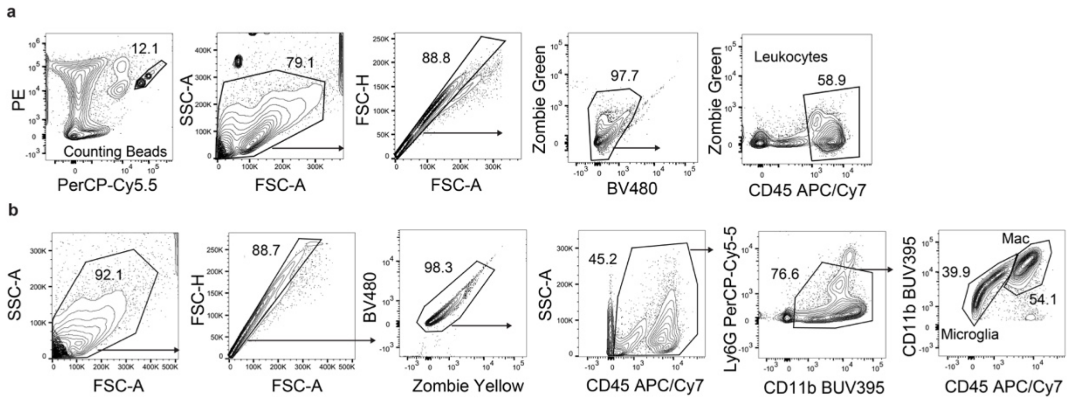

Supplementary Data Fig. 3 | Characterization of microglia and CNS-infiltrating immune cells. EAE was induced in *Gdf15*-proficient and -deficient mice. Immune cells were isolated from the brain and spinal cord during acute EAE (day 15 p.i.): **a**, Representative gating strategy for quantification of leukocytes. **b**, Representative gating strategy for the characterization of microglia and infiltrating macrophages.

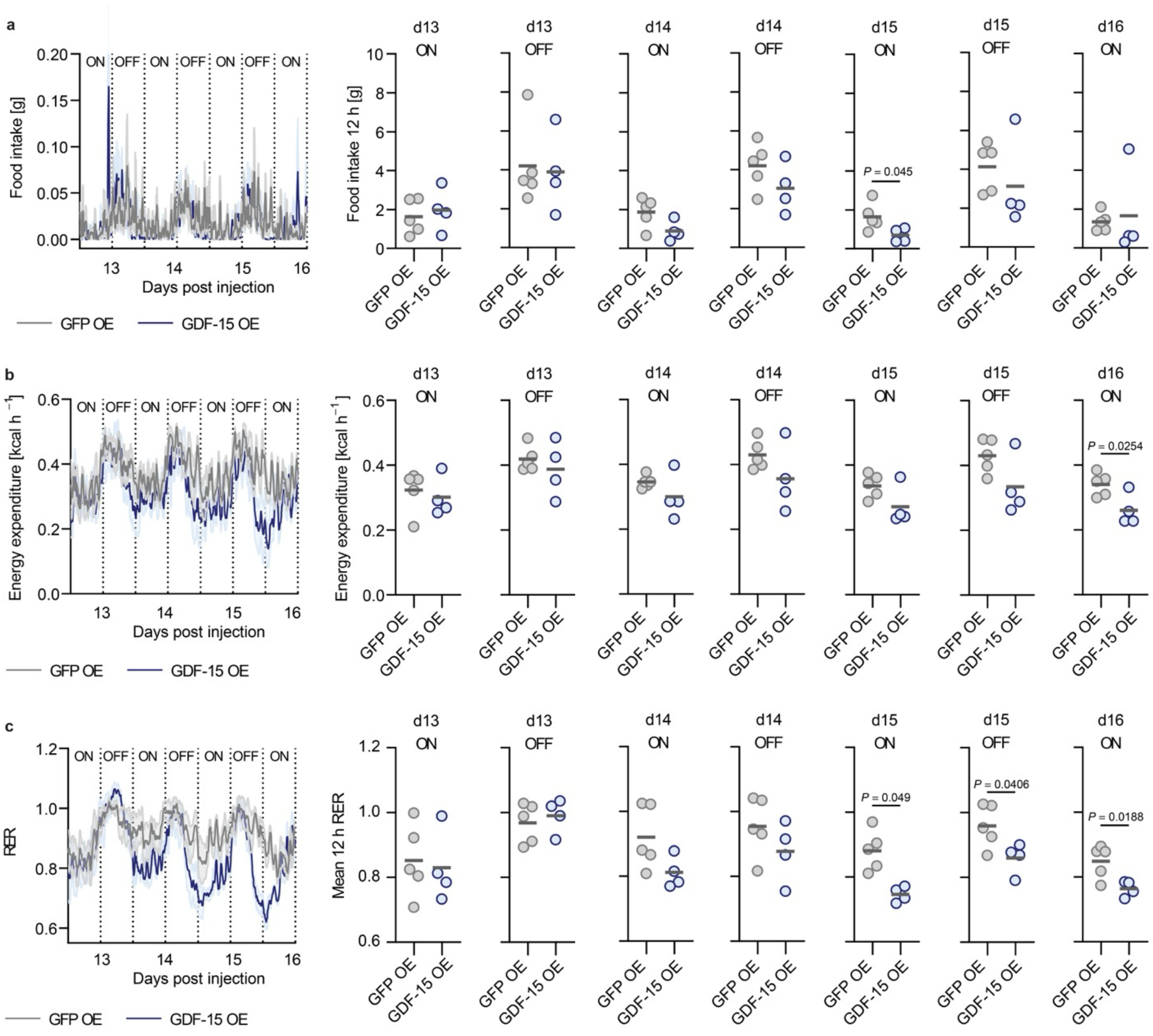

Supplementary Data Fig. 4 | Neuronal GDF-15 delivery drives metabolic adaptation. a-c, Female C57BL/6J mice were injected with 1 × 10^{11} vg per animal of a an rAAV coding for eGFP (GFP OE, *n* = 5) or mouse GDF-15 (GDF-15 OE, *n* = 4). Mice were continuously monitored in individual metabolic cages. **a**, Food intake, **b**, energy consumption, and **c**, respiratory exchange ratio (RER). Quantification was performed for 12-hours intervals of light (ON) and dark (OFF) phases. Individual datapoints represent biological replicates. Data are shown as mean ± SEM (**a-c**). For (**a-c**), unpaired two-sided *t*-tests were performed. OE = overexpression.

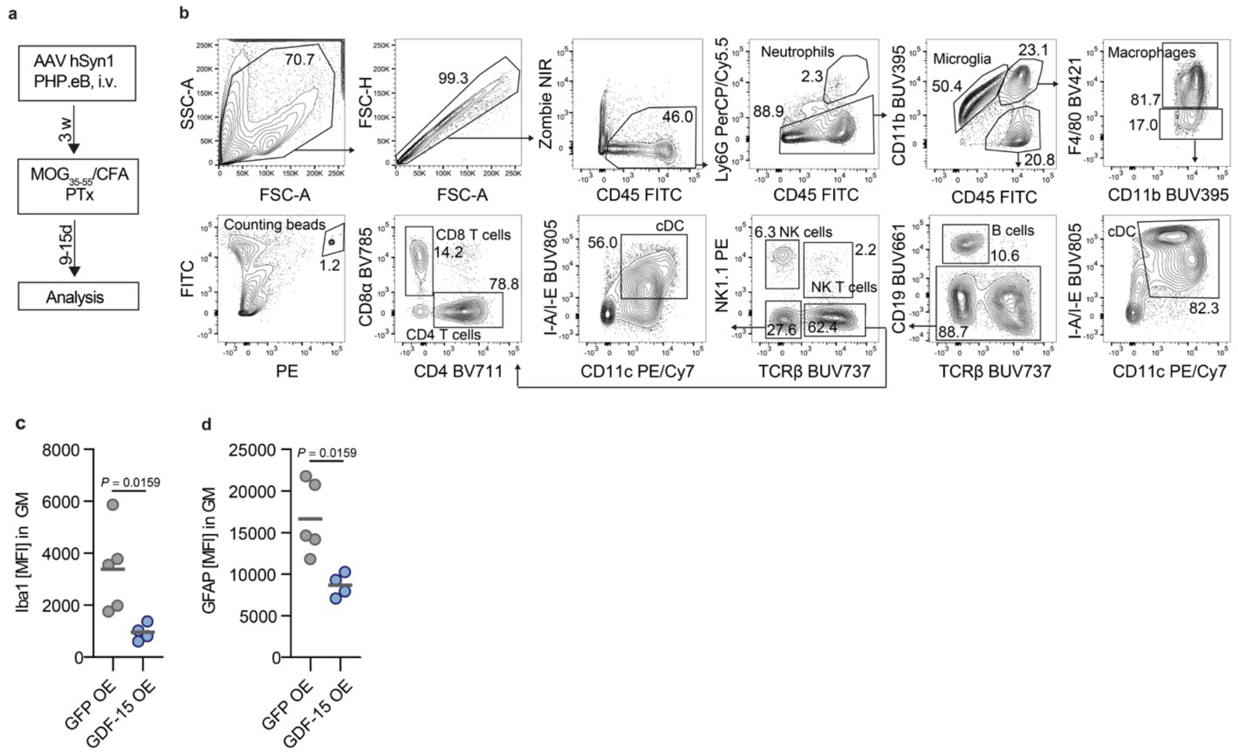

Supplementary Data Fig. 5 | Analysis of immune cell infiltration and glia activation after neuronal GDF-15 delivery. **a**, Scheme for rAAV-mediated gene delivery of GFP (GFP OE) or mouse GDF-15 (GDF-15 OE) prior to EAE induction. Analyses were performed during acute EAE (day 15 post immunization (p.i.). **b**, Representative gating strategy for the quantification of immune cell infiltrates in spinal cord tissue of EAE animals. Precision Count beads are used to determine absolute cell counts. **c**, Mean fluorescence intensity (MFI) of Iba1 in the grey matter (GM) of cervical spinal cord sections in acute EAE; n = 5 for GFP OE, n = 4 for GDF-15 OE. **d**, MFI of GFAP in the GM of cervical spinal cord sections in acute EAE (day 15 p.i.); n = 5 for GFP OE, n = 4 for GDF-15 OE. Individual datapoints represent biological replicates. In (**c, d**), unpaired two-sided Mann-Whitney tests were performed. OE = overexpression.

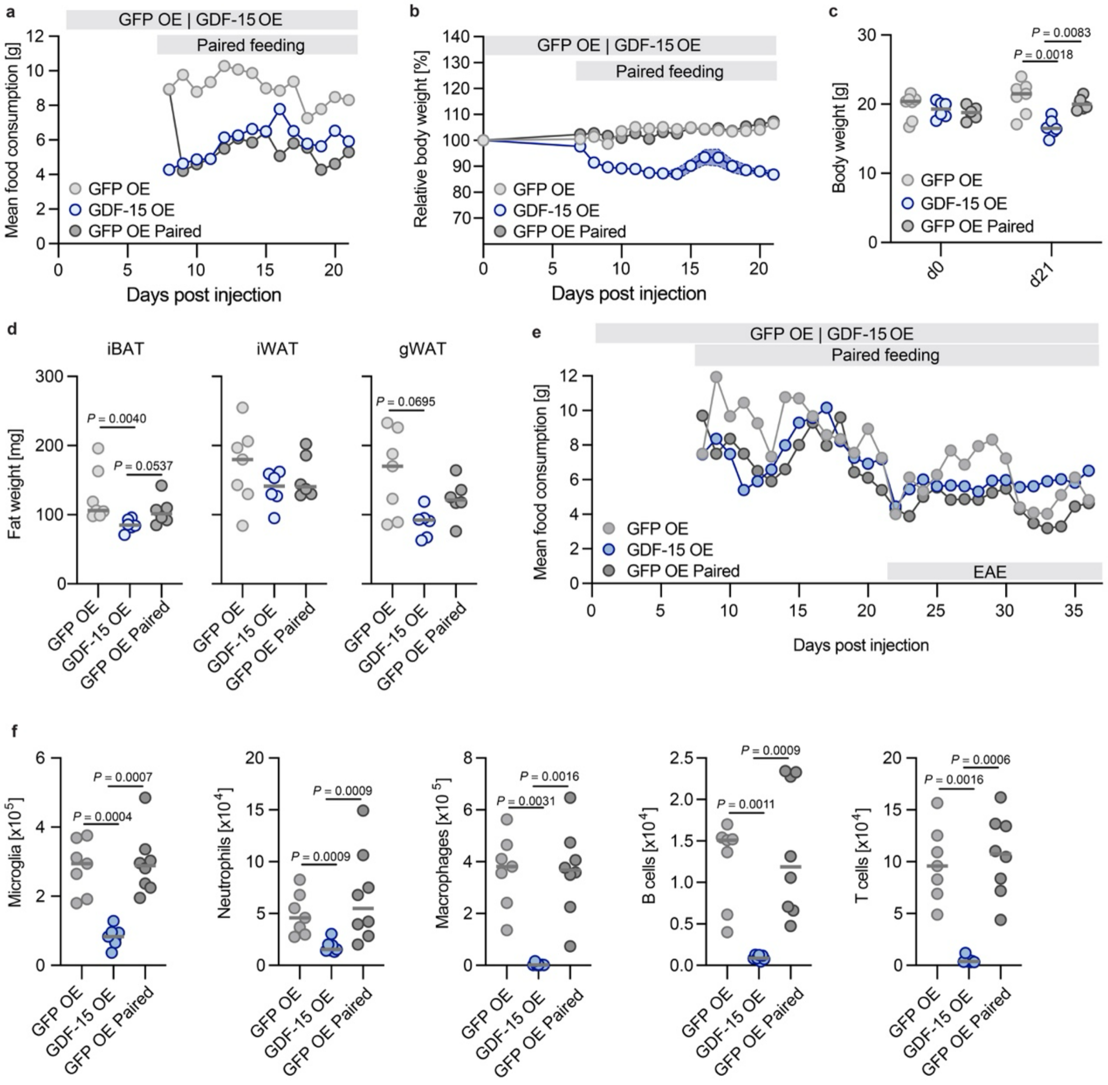

Supplementary Data Fig. 6 | Caloric restriction does not phenocopy protective effects of *Gdf15* delivery. a-d, Female C57BL/6J mice were injected with an rAAV coding for eGFP (GFP OE) or mouse GDF-15 (GDF-15 OE). The GFP OE pair-fed group received restricted amounts of food based on the intake of GDF-15 OE mice; $n = 7$ per group. **a**, Mean food consumption in 24 hours interval. **b**, Relative body weight loss. **c**, Absolute body weight on day 0 and day 21 post-injection. **d**, Total weight of interscapular brown adipose tissue (iBAT), inguinal white adipose tissue (iWAT) and gonadal white adipose tissue (gWAT) on day 21 post-injection. **e-f**, Female C57BL/6J mice were injected with an rAAV coding for eGFP (GFP OE) or eGFP and mouse GDF-15 (GDF-15 OE); $n = 7$ for GFP OE and GDF-15 OE, $n = 8$ for GFP OE Paired. **e**, Mean food consumption in 24 hours interval. **f**, Absolute numbers of microglia, neutrophils, macrophages, B cells and T cells in spinal cord tissue quantified by flow cytometry in acute EAE (day 15 p.i.). Individual datapoints represent biological replicates. Data are shown as mean (**a**, **e**), or mean ± SEM (**b**). For (**c**, **d**, **f**), a Kruskal-Wallis test with FDR correction was performed. OE = overexpression.

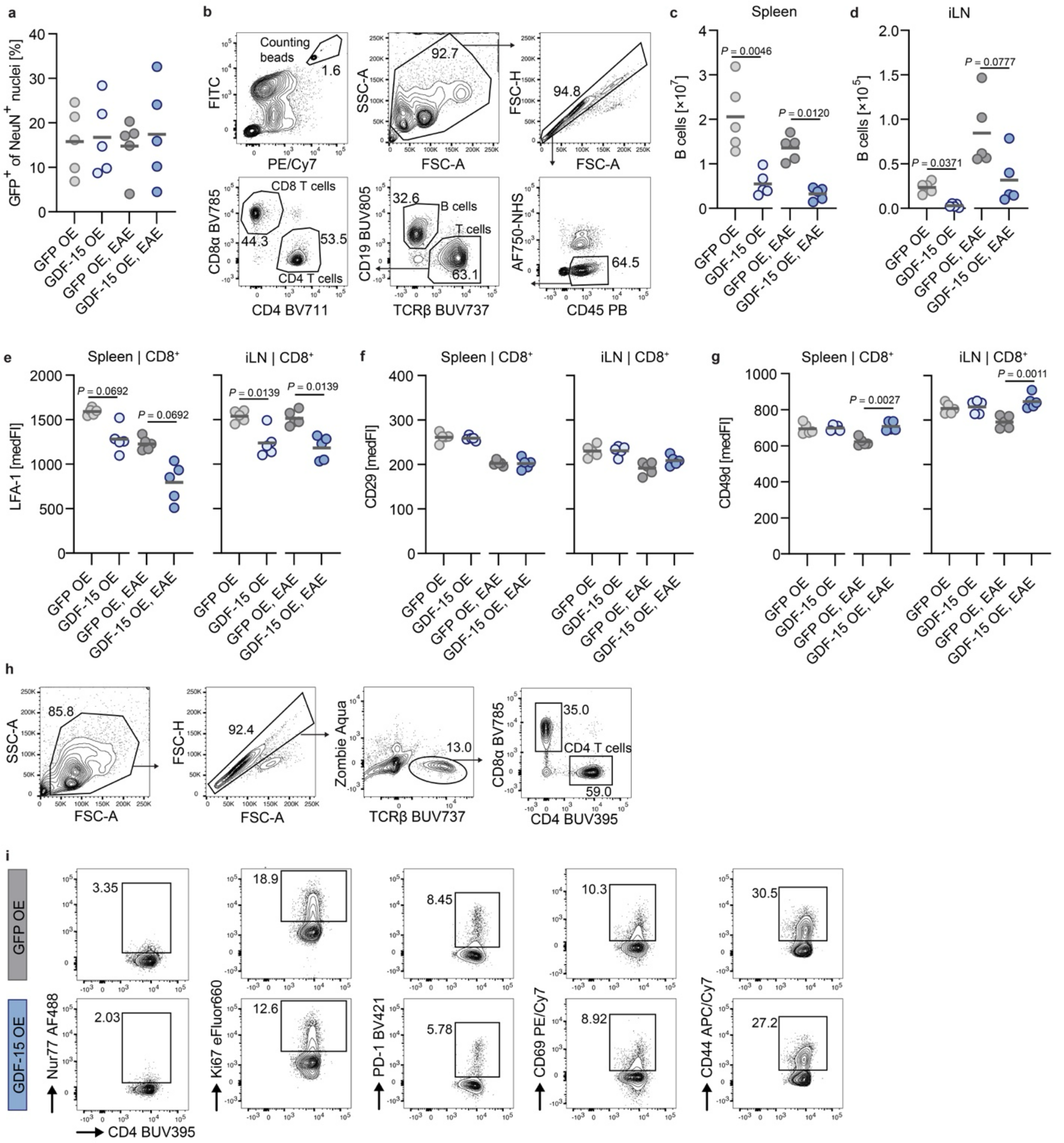

Supplementary Data Fig. 7 | GDF-15 delivery alters peripheral lymphocyte phenotype. a-g,Female C57BL/6J mice were injected with 1 × 10^{11} vg per animal of a PHP.eB AAV coding for eGFP (GFP OE) or mouse GDF-15 (GDF-15 OE). In preclinical EAE groups, mice were immunized, and organs were collected on day 9 post immunization (p.i.) prior to disease onset; *n* = 5 per group. **a**, Transduction efficacy in thoracic spinal cord tissue. **b**, Representative gating strategy for the quantification of immune cell subsets. **c**, Absolute numbers of B cells in the spleen. **d**, Absolute numbers of B cells in the inguinal lymph nodes (iLN). **e-g**, Median fluorescence intensity (medFI) of selected markers on $CD8^+$ T cells. **e**, LFA-1 in the spleen (left) and iLN (right). **f**, CD29 in the spleen (left) and iLN (right). **g**, CD49d in the spleen (left) and iLN (right). **h**, Representative gating strategy for the quantification of selected markers in $CD4^+$ T cells in the spleen from preclinical EAE animals after rAAV delivery. **i**, Representative contour plots to quantify the frequency of $Nur77^+$, $Ki67^+$, programmed death-1 $(PD-1)^+$, $CD69^+$ and $CD44^{hi}$ in $CD4^+$ T cells in the spleen. Individual datapoints represent biological replicates. For (**c-g**) two-sided Mann-Whitney tests were performed. OE = overexpression.

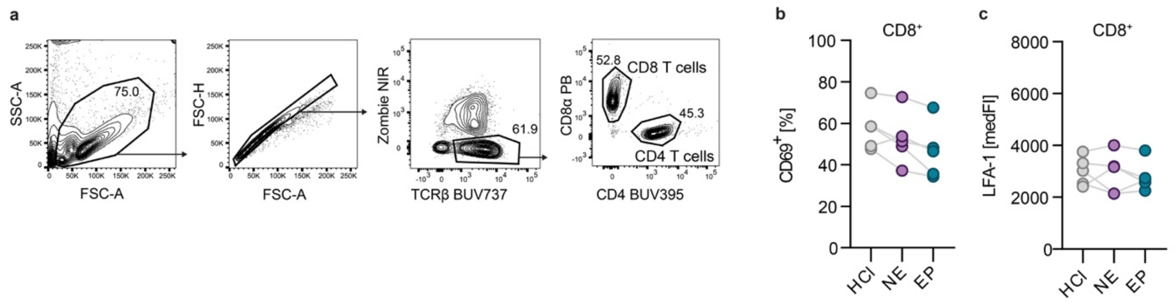

Supplementary Data Fig. 8 | Activation of β-adrenoceptors has no effect on CD8⁺ T cell activation. a, Representative gating strategy for primary mouse T cells stimulated with β-adrenergic receptor agonists or antagonists. **b**, **c**, T cells were isolated from spleen and inguinal lymph nodes (iLN) from female mice. Cells were stimulated with anti-CD3/CD28 and endogenous agonists of β-adrenergic receptors; n = 5. **b**, Frequency of CD69⁺ cells within total CD8⁺ T cells stimulated with 10 μM norepinephrine (NE), 10 μM epinephrine (EP) or HCl after 24 hours. **c**, Median fluorescence intensity (medFI) of LFA-1 on total CD8⁺ T cells stimulated with 10 μM NE, 10 μM EP or HCl after 24 hours. Individual datapoints represent biological replicates. For (**b**, **c**) two-sided Mann-Whitney tests were performed.

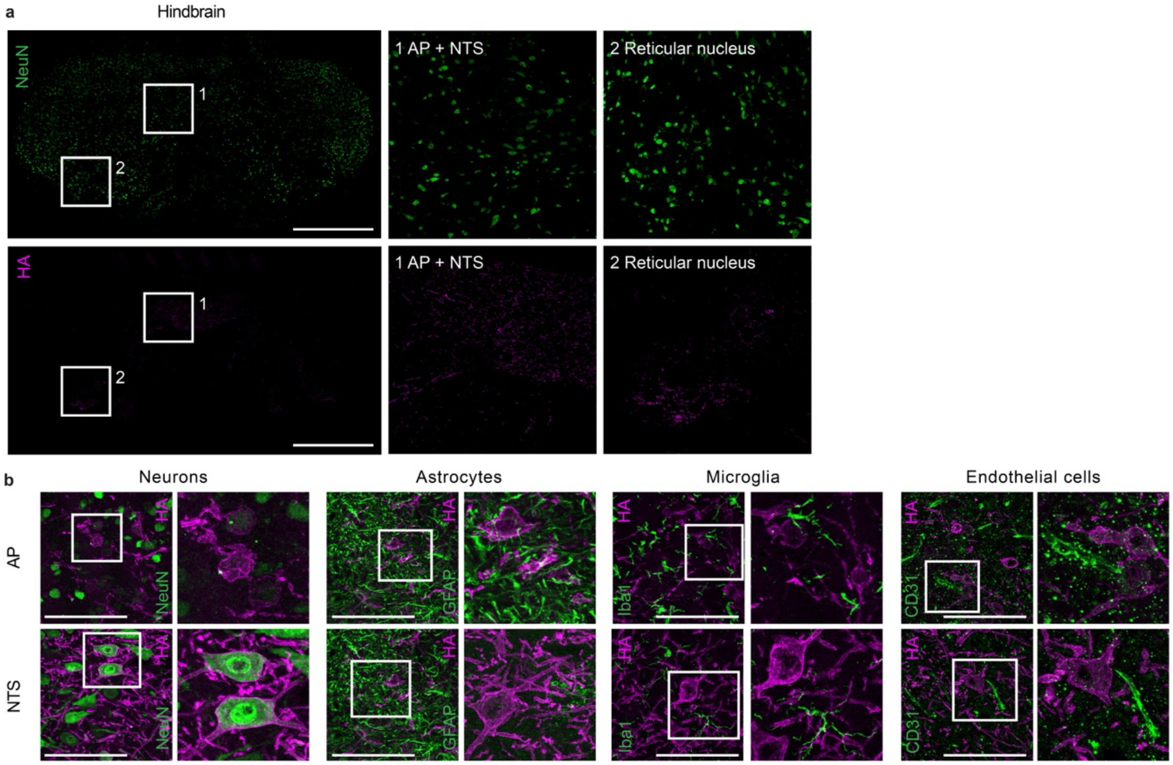

Supplementary Data Fig. 9 | Expression of hM3Dq is restricted to neurons. a, Expression of HA (hM3Dq) in coronal hindbrain sections from GFRAL-Cre × LSL-hM3Dq-DREADD mice. Scale bar shows 1 mm. **b**, Z-stacks of area postrema (AP; upper row) and nucleus tractus solitarius (NTS, lower row) sections showing HA (hM3Dq)$^{+}$ cells co-stained with markers for neurons (NeuN^{+}), astrocytes (GFAP^{+}), microglia (Iba1^{+}), and endothelial cells (CD31^{+}). Scale bar shows 200 µm. In (**a, b**) images from one representative animal are shown.

SUPPLEMENTARY TABLES

Supplementary Table 1 | Demographics of pregnant women and non-pregnant controls.

Group	*n* (% female)	Age, years, mean (SD)	BMI, mean (SD)
Control	13 (100)	30.4 (4.3)	23.9 (4.1)
Pregnant	13 (100)	30.8 (4.1)	23.8 (4.2)

Supplementary Table 2 | Demographics of women with abortion or miscarriage.

Group	*n* (% female)	Age, years, mean (SD)	BMI, mean (SD)
Abortion	10 (100)	25.2 (5.3)	23.4 (1.5)
Miscarriage	10 (100)	29.8 (7.5)	23.4 (1.9)

Supplementary Table 3 | Demographics of pregnant MS patients.

Group	*n* (% female)	Age, years, mean (SD)	BMI, mean (SD)
Stable	58 (100)	31.7 (4.2)	24.5 (4.7)
Relapse	12 (100)	29.4 (4.8)	23.8 (3.5)

Supplementary Table 4 | Demographics of MS patients and controls.

**Control vs. progressive MS (PMS)**				
**Group**	**Diagnosis (% total)**	***n* (% female)**	**Age, years, mean (SD)**	**Treatment, *n* (%)**
Control	NA	18 (72.2)	50.8 (6.2)	0 (0)
PMS	PPMS (55.6 %)	20 (45.0)	54.2 (5.2)	0 (0)
**Relapse vs. remission**				
**Group**	**Timepoint**	***n* (% female)**	**Age, years, mean (SD)**	**Treatment, *n* (%)**
RRMS	Remission	8 (62.5)	29.3 (6.2)	0 (0)
