# Supplementary material for: A GDF-15–GFRAL axis controls autoimmune T cell responses during neuroinflammation
Source: Nat Immunol. 2026 Jan 15;27(3):503–15. doi: 10.1038/s41590-025-02406-1 (PMC12956584; doi:10.1038/s41590-025-02406-1)
Supplement: Supplementary file 2 — Reporting Summary [file 41590_2025_2406_MOESM2_ESM.pdf]

nature portfolio

Corresponding author(s): Manuel Friese

Last updated by author(s): Nov 18, 2025

Reporting Summary

Nature Portfolio wishes to improve the reproducibility of the work that we publish. This form provides structure for consistency and transparency in reporting. For further information on Nature Portfolio policies, see our Editorial Policies and the Editorial Policy Checklist.

Statistics

For all statistical analyses, confirm that the following items are present in the figure legend, table legend, main text, or Methods section.

n/a	Confirmed	
☐	☒	The exact sample size (*n*) for each experimental group/condition, given as a discrete number and unit of measurement
☐	☒	A statement on whether measurements were taken from distinct samples or whether the same sample was measured repeatedly
☐	☒	The statistical test(s) used AND whether they are one- or two-sided<br>*Only common tests should be described solely by name; describe more complex techniques in the Methods section.*
☒	☐	A description of all covariates tested
☐	☒	A description of any assumptions or corrections, such as tests of normality and adjustment for multiple comparisons
☐	☒	A full description of the statistical parameters including central tendency (e.g. means) or other basic estimates (e.g. regression coefficient) AND variation (e.g. standard deviation) or associated estimates of uncertainty (e.g. confidence intervals)
☐	☒	For null hypothesis testing, the test statistic (e.g. *F*, *t*, *r*) with confidence intervals, effect sizes, degrees of freedom and *P* value noted<br>*Give P values as exact values whenever suitable.*
☒	☐	For Bayesian analysis, information on the choice of priors and Markov chain Monte Carlo settings
☒	☐	For hierarchical and complex designs, identification of the appropriate level for tests and full reporting of outcomes
☒	☐	Estimates of effect sizes (e.g. Cohen's *d*, Pearson's *r*), indicating how they were calculated

Our web collection on statistics for biologists contains articles on many of the points above.

Software and code

Policy information about availability of computer code

Data collection	Flow cytometric nuclei or cell sorting: BD FACSDiva v9.0.1 software (BD Bioscience) to run BD FACS Aria III.<br>Flow cytometry data collection: FACSDiva software version 9.1 software (BD Biosciences) to run BD Symphony A3.<br>Bulk RNAsequencing: NovaSeq6 000 platform (Illumina).<br>Confocal imaging: Zeiss LSM 900 Airyscan 2 confocal microscope equipped with ZEN blue software v3.9.<br>Immunoblots: LAS4000 Image-Reader software.<br>RT-qPCR: QuantStudio Flex 6 Real-Time PCR System.<br>Metabolomics: WebIDQ cloud-based software package (BIOCRATES, version 2024).
Data analysis	Image analysis fluorescence microscopy and immunoblots: ImageJ 1.54i<br>Flow cytometry: FlowJo version 10.9 (BD Biosciences)<br>Bulk RNAsequencing: STAR v2.7.9a, featureCounts v1.5.1, DESeq2 v1.40.2, biomaRt v2.56.1, ggplot2 v3.4.3.<br>Data representation and statistics: Prism 10.4.2 (GraphPad).<br>Metabolomics: WebIDQ cloud-based software package (BIOCRATES, version 2024).<br>Power analysis: G*Power 3.1<br>RT-qPCR: Thermo Fisher Cloud

For manuscripts utilizing custom algorithms or software that are central to the research but not yet described in published literature, software must be made available to editors and reviewers. We strongly encourage code deposition in a community repository (e.g. GitHub). See the Nature Portfolio guidelines for submitting code & software for further information.

Data

Policy information about availability of data

All manuscripts must include a data availability statement. This statement should provide the following information, where applicable:

- Accession codes, unique identifiers, or web links for publicly available datasets
- A description of any restrictions on data availability
- For clinical datasets or third party data, please ensure that the statement adheres to our policy

Sequencing data generated for this study are available through the Gene Expression Omnibus under accession number GSE288193. All other data are available in the main text or the supplementary materials.

Research involving human participants, their data, or biological material

Policy information about studies with human participants or human data. See also policy information about sex, gender (identity/presentation), and sexual orientation and race, ethnicity and racism.

Reporting on sex and gender	We report the sex of all participants in Supplementary Table 1 to 4.
Reporting on race, ethnicity, or other socially relevant groupings	For this study, we selected participants blind to their age and ethnicity. Most participants were female since we study the dynamics of GDF-15 in pregnancy. The demographic characteristics are described in Supplementary Table 1 to 4.
Population characteristics	For this study, we selected participants blind to their age ethnicity based on the availability of frozen serum samples. The demographic characteristics are described in Supplementary Table 1 to 4.
Recruitment	MS patients and healthy individuals were recruited through the MS outpatient clinic of the Department of Neurology, University Medical Center Hamburg-Eppendorf.<br>Pregnant women were recruited through the PRINCE (PRenatal IdeNtification of Children's HEalth) study which enrolled women of legal age experiencing a singleton pregnancy during their first trimester (gestational weeks 12–14).<br>Serum samples of pregnant women who experienced miscarriage or performed elective abortion were collected during routine blood sampling and processed according to standard laboratory methods at the Laboratory for Pediatric Rheumatology/Special Immunology at the University Hospital Wuerzburg.<br>Participants did not receive any financial compensation.
Ethics oversight	MS patients and healthy individuals: Hamburg Chamber of Commerce Act for the Health Professions, registration number PV4405.<br>Pregnant women recruited through the PRINCE: Hamburg Chamber of Physicians (license number PV3694).<br>Pregnant women who experienced miscarriage or performed elective abortion: ethics protocol numbers 28/08 and 239/10.<br>The study adhered to the principles outlined in the Declaration of Helsinki for medical research involving human subjects.

Note that full information on the approval of the study protocol must also be provided in the manuscript.

Field-specific reporting

Please select the one below that is the best fit for your research. If you are not sure, read the appropriate sections before making your selection.

☒ Life sciences ☐ Behavioural & social sciences ☐ Ecological, evolutionary & environmental sciences

For a reference copy of the document with all sections, see nature.com/documents/nr-reporting-summary-flat.pdf

Life sciences study design

All studies must disclose on these points even when the disclosure is negative.

Sample size	Sample sizes were estimated based on previous extensive experience in the laboratory with the EAE model. For in vitro experiments with primary cells at least 4 biological replicates were used based on 3R principles, sample sizes of previous published studies and the fact that experiments were performed in inbred mice.
Data exclusions	In the EAE model, mice without ulcerations at the injection site prior to disease onset were excluded from the analyses. For immunohistochemical analysis specimens with impaired tissue integrity were removed from the analysis. No samples were excluded from the analysis based on outlier detection. For the TH immunoblot in DREADD animals the last specimen was excluded from analysis due to incomplete transfer to the membrane.
Replication	EAE experiments were performed twice (Gdf15-/-) with different readouts or repeated multiple times with adapted strategies (GDF-15 OE, combined i.e. with paired feeding or mutant GDF-15). All attempts to replicate findings were successful. Experiments with Gfral-/- and DREADD animals were only performed once, but with sufficient power to detect differences.<br>All data derived from primary cells/tissues were performed with biological replicates. Experiments with T cells treated with ADRB agonists and antagonists were performed at least twice with similar results.

For experiments with cell lines at least three independent experiments were performed with similar results, unless stated otherwise.

Randomization	In EAE experiments, mice were randomly assigned to treatment or control groups (Control vs effector AAV, treatment vs vehicle). These mice were mixed within cages to minimize cage-specific effects. Only for paired-feeding experiments animals belonging to the same group were housed together in groups of 2-3 animals per cage since food intake had to monitored for each group separetely. For experiments with transgenic mice (Gdf15-/-, Gfral-/-, DREADD) cages contained littermates of different genotypes to minimize cage-effects.
Blinding	Mouse scoring was conducted in a blinded manner, with researchers not aware of genotype, the injected AAV or the treatment to prevent observer bias. Since for the paired-feeding experiments animals belonging to different experimental groups had to be housed separately (see randomization), one researcher monitored food consumption, while a second independent researcher assessed the clinical score. For all downstream analyses (histology, flow cytometry, etc.) the reseacher was blinded during acquisition and analysis. The analysis of plasma or serum samples was performed in a blinded manner as the researcher measuring GDF-15 or catecholamines was not aware of the group assignment.

Reporting for specific materials, systems and methods

We require information from authors about some types of materials, experimental systems and methods used in many studies. Here, indicate whether each material, system or method listed is relevant to your study. If you are not sure if a list item applies to your research, read the appropriate section before selecting a response.

Materials & experimental systems

n/a	Involved in the study	
☐	☒	Antibodies
☐	☒	Eukaryotic cell lines
☒	☐	Palaeontology and archaeology
☐	☒	Animals and other organisms
☒	☐	Clinical data
☒	☐	Dual use research of concern
☒	☐	Plants

Methods

n/a	Involved in the study	
☒	☐	ChIP-seq
☐	☒	Flow cytometry
☒	☐	MRI-based neuroimaging

Antibodies

Antibodies used

ACSA-2 APC Miltenyi Biotec 130-117-535 Rat IH3-18A3 1:50 IF, IHC, FC
BTLA PE BioLegend 139107 Armenian Hamster 6A6 1:100 FC - Quality tested
CD3 BioLegend 100301 Armenian Hamster 145-2C11 1:200 FC - Quality tested
CD4 Pacific Blue BioLegend 100531 Rat RM4-5 1:400 FC - Quality tested
CD4 BUV395 BD Bioscience 563790 Rat GK1.5 1:200 FC (Routinely Tested)
CD4 BV711 BioLegend 100447 Rat GK1.5 1:200 FC - Quality tested
CD8α BV785 BioLegend 100750 Rat 53-6.7 1:200 FC - Quality tested
CD8α Pacific Blue BioLegend 100725 Rat 53-6.7 1:400 FC - Quality tested
CD11b BUV395 BD Bioscience 563553 Rat M1/70 1:400 FC (Routinely Tested)
CD11b BV785 BioLegend 101243 Rat M1/70 1:200 FC - Quality tested
CD11c PE-Cy7 BioLegend 117318 Rat N418 1:400 FC - Quality tested
CD19 BUV661 BD Bioscience 612971 Rat 1D3 1:400 FC (Routinely Tested)
CD19 BUV805 BD Bioscience 568287 Rat 1D3 1:400 FC (Routinely Tested)
CD19 PE-Cy7 BioLegend 115519 Rat 6D5 1:400 FC - Quality tested
CD25 PE BioLegend 102008 Rat PC61 1:100 FC - Quality tested
CD29 PE-Cy7 BioLegend 102222 Armenian Hamster HMβ1-1 1:100 FC - Quality tested
CD31 R&D systems AF3628 Goat polyclonal 1:100 ELISA, WB, ICH, ICC, FC
CD44 APC-Cy7 BioLegend 103028 Rat IM7 1:200 FC - Quality tested
CD44 PerCP-Cy5.5 BioLegend 103032 Rat IM7 1:200 FC - Quality tested
CD45 BioLegend 103101 Rat 30-F11 1:200 FC - Quality tested
CD45 APC-Cy7 BioLegend 103116 Rat 30-F11 1:200 FC - Quality tested
CD45 FITC BioLegend 103108 Rat 30-F11 1:200 FC - Quality tested
CD45 Pacific Blue BioLegend 103126 Rat 30-F11 1:200 FC - Quality tested
CD49d PE BioLegend 103607 Rat R1-2 1:50 FC - Quality tested
CD68 BioLegend 137002 Rat FA-11 1:1000 FC - Quality tested
CD69 PE-Cy7 BioLegend 104512 Armenian Hamster H1.2F3 1:200 FC - Quality tested
CD127 BV785 BioLegend 135037 Rat A7R34 1:100 FC - Quality tested
CX3CR1 Pacific Blue BioLegend 149038 Mouse SA011F11 1:200 FC - Quality tested
F4/80 BV421 BioLegend 123132 Rat BM8 1:100 FC - Quality tested
GDF-15 Evitria custom-made Mouse 297 20 µg/ml N/A
GFAP Merck Millipore AB5541 Chicken polyclonal 1:800 1:800 ICC, IHC, WB
GFP Abcam ab13970 Chicken polyclonal 1:2000 WB, ICC/IF
GPNMB eFluor660 Invitrogen 50-5708-80 Rat CTSREVL 1:50 FC
HA Sigma-Aldrich 11867423001 Rat 3F10 1:400 ELISA, IHC, IP, WB
I-A/I-E BUV805 BD Bioscience 748844 Rat M5/114.15.2 1:200 FC (Routinely Tested)
Iba1 Synaptic Systems 234308 Guinea Pig Gp311H9 1:500 WB, IP, ICC, IHC

Iba1 Wako 019-19741 Rabbit polyclonal 1:1000 ICC, IHC
LFA-1 APC BioLegend 141010 Rat H155-78 1:100 FC - Quality tested
Ki-67 eFluor 660 eBioscience 50-5698-80 Rat SolA15 1:50 IHC, ICC, FC
Ly6C PE BioLegend 128008 Rat HK1.4 1:200 FC - Quality tested
Ly6G PerCP-Cy5.5 BioLegend 127616 Rat 1A8 1:100 FC - Quality tested
NeuN Millipore ABN91 Chicken polyclonal 1:400 WB, ICC, IHC
NeuN Synaptic Systems 266004 Guinea Pig polyclonal 1:250 1:250 ICC, IHC
NeuN Alexa Fluor 647 Abcam ab190565 Rabbit EPR12763 1:500 IHC, ICC
Nk1.1 APC BioLegend 108710 Mouse PK136 1:100 FC - Quality tested
NK1.1 PE Invitrogen 12-5941-82 Mouse PK136 1:200 FC
NK1.1 PE-Cy7 BioLegend 108714 Mouse PK136 1:200 FC - Quality tested
Nur77 AF488 Invitrogen 53-5965-82 Mouse 12.14 1:50 FC
P2RY12 PE BioLegend 848004 Rat S16007D 1:100 FC - Quality tested
PD-1 (CD279) BV421 BioLegend 109121 Rat RMP1-30 1:100 FC - Quality tested
pErk1/2 BioLegend 369516 Mouse 6B8B69 1:20 ICFC - Quality tested
RFP ChromoTek 5f8 Rat 5F8 1:1000 IF, ELISA
RFP Rockland R10367 Rabbit polyclonal 1:1000 ELISA, IF, IHC, WB
Tubulin-β3 BioLegend 801213 Mouse TUJ1 1:200 IHC-P, WB, ICC
TCRβ BUV737 BD Bioscience 612821 Armenian Hamster H57-597 1:100 FC (Routinely Tested)
TCRβ BV421 BioLegend 109230 Armenian Hamster H57-597 1:200 FC - Quality tested
Tyrosine hydroxylase Proteintech 25859-1-AP Rabbit polyclonal 1:5000 WB, IP, IHC, IF
Tyrosine hydroxylase Sigma-Aldrich AB152 Rabbit polyclonal 1:500 ELISA, IF, IHC, IP, WB
Vinculin Merck V9131 Mouse hVin-1 1:1000 WB, IF, IHC

Donkey Ig chicken Alexa Fluor 488 Jackson Immuno 703-545-155 Donkey polyclonal 1:500 ICC, IHC
Donkey Ig chicken Alexa Fluor Cy3 Jackson Immuno 703-165-155 Donkey polyclonal 1:500 ICC, IHC
Donkey Ig chicken Alexa Fluor 647 Jackson Immuno 703-606-155 Donkey polyclonal 1:500 ICC, IHC
Donkey Ig goat Alexa Fluor 488 Abcam ab150129 Donkey polyclonal 1:500 ELISA, IHC-Fr, IHC-P, FC, ICC/IF
Donkey Ig goat Alexa Fluor 555 Abcam ab150130 Donkey polyclonal 1:500 ELISA, IHC-Fr, IHC-P, FC, ICC/IF
Donkey Ig guinea pig Alexa Fluor 488 Jackson Immuno 706-545-148 Donkey polyclonal 1:500 ICC, IHC
Donkey Ig guinea pig Alexa Fluor Cy3 Jackson Immuno 706-165-148 Donkey polyclonal 1:500 ICC, IHC
Donkey Ig guinea pig Alexa Fluor 647 Jackson Immuno 706-605-148 Donkey polyclonal 1:500 ICC, IHC
Donkey Ig rabbit Alexa Fluor 488 Abcam ab150073 Donkey polyclonal 1:500 ELISA, IHC-Fr, IHC-P, FC, ICC/IF
Donkey Ig rabbit Alexa Fluor 555 Abcam ab150062 Donkey polyclonal 1:500 ELISA, IHC-Fr, IHC-P, FC, ICC/IF
Donkey Ig rat Alexa Fluor 555 Abcam ab150150 Donkey polyclonal 1:500 ELISA, IHC-Fr, IHC-P, FC, ICC/IF
Donkey Ig rabbit Alexa Fluor 647 Abcam ab181347 Donkey polyclonal 1:500 ELISA, IHC-Fr, IHC-P, FC, ICC/IF
Donkey Ig rat Alexa Fluor 647 Abcam ab150151 Donkey polyclonal 1:500 ELISA, IHC-Fr, IHC-P, FC, ICC/IF
Donkey Ig Mouse Alexa Fluor 647 Abcam ab150111 Donkey polyclonal 1:500 ELISA, IHC-Fr, IHC-P, FC, ICC/IF
DyLight 649 IgG Armenian hamster BioLegend 405505 Goat polyclonal 1:200 FC - Quality tested

Goat anti-mouse HRP LICORbio 926-80010 Goat polyclonal 1:10000 WB
Goat anti-rabbit HRP Cell Signaling 7074 Goat polyclonal 1:5000 WB, IHC, ELISA
Goat anti-rat HRP Cell Signaling 7077 Goat polyclonal 1:5000 WB, IHC, ELISA

Validation

Antibody validation for the species is provided for all commercially available antibodies on the relevant manufacturer's website accessible via the catalogue numbers which are provided above.

Eukaryotic cell lines

Policy information about cell lines and Sex and Gender in Research

Cell line source(s)

HEK 293T (ACC 635, DMSZ, purchased in 2017), Neuro-2a (ACC 148, DSMZ, purchased 2022), SIM-A9 (T0247-GVO-ABM, Biocat, purchased 2024).

Authentication

All cell lines used in this study were purchased from authorized vendors.

Mycoplasma contamination

All cell lines were regularly checked for mycoplasma contamination using the VenorGeM Advance kit (Minerva biolabs, 11-7024) according to the manufacturer's instructions. Cell lines were free of mycoplasma contamination.

Commonly misidentified lines
(See ICLAC register)

None of the cell lines used in this study is listed in the registry.

Animals and other research organisms

Policy information about studies involving animals; ARRIVE guidelines recommended for reporting animal research, and Sex and Gender in Research

Laboratory animals

All mice (C57BL/6J and BALB/c wild-type purchased from Charles River; Gdf15–/–; Gfral–/–, Gfral-Cre (purchased from the Jackson Laboratory, #036750), Ai14, LSL-hM3Dq-DREADD, Gfral-Cre × Ai14, Gfral-Cre × LSL-hM3Dq-DREADD, Adrb2–/– were kept under specific pathogen-free conditions in the central animal facility of the University Medical Center Hamburg-Eppendorf. Adult mice (6–20 weeks old) from both sexes were used, unless otherwise stated; mice were sex- and age-matched in all experiments. The mice were kept in a 12-hour light/dark diurnal cycle, 22 ± 2 °C, 40-60 % humidity and given ad libitum access to standard chow (Altromin, 1328P) and water, unless otherwise stated. EAE mice additionally received DietGel® Recovery (Ssniff; H007-72065).
We thank Jens Strelau (Department of Functional Neuroanatomy, University of Heidelberg, Heidelberg, Germany) for providing

Gdf15–/– animals, Christine Gee Gee (Institute for Synaptic Neuroscience, ZMNH, University Medical Center Hamburg-Eppendorf, Hamburg, Germany) for providing LSL-hM3Dq-DREADD animals, Ora Ohana for providing Ai14 animals (Institute for Molecular and Cellular Cognition, ZMNH, University Medical Center Hamburg-Eppendorf, Hamburg, Germany) to generate GFRAL reporter animals, and Johannes Keller and Anke Baranowsky (Department of Trauma and Orthopaedic Surgery, University Medical Center Hamburg-Eppendorf, Hamburg, Germany) for providing Adrb2–/– animals.

Wild animals

This study does not involve wild animals.

Reporting on sex

For EAE experiments involving C57BL/6J WT mice, only female animals were included. For all other experiments the sex is specified in the figure legend.

Field-collected samples

This study does not include field-collected samples.

Ethics oversight

All animal care and experimental procedures were conducted in accordance with institutional guidelines and met the requirements of the German legal authorities. Ethical approvals were obtained from the State Authority of Hamburg, Germany (Behörde für Justiz und Verbraucherschutz, Freie und Hansestadt Hamburg; approval no. 45/17, N007/22, N108/24).

Note that full information on the approval of the study protocol must also be provided in the manuscript.

Plants

Seed stocks

No plants were uses in this study

Novel plant genotypes

Describe the methods by which all novel plant genotypes were produced. This includes those generated by transgenic approaches, gene editing, chemical/radiation-based mutagenesis and hybridization. For transgenic lines, describe the transformation method, the number of independent lines analyzed and the generation upon which experiments were performed. For gene-edited lines, describe the editor used, the endogenous sequence targeted for editing, the targeting guide RNA sequence (if applicable) and how the editor was applied.

Authentication

Describe any authentication procedures for each seed stock used or novel genotype generated. Describe any experiments used to assess the effect of a mutation and, where applicable, how potential secondary effects (e.g. second site T-DNA insertions, mosiacism, off-target gene editing) were examined.

Flow Cytometry

Plots

Confirm that:

☒ The axis labels state the marker and fluorochrome used (e.g. CD4-FITC).

☒ The axis scales are clearly visible. Include numbers along axes only for bottom left plot of group (a 'group' is an analysis of identical markers).

☒ All plots are contour plots with outliers or pseudocolor plots.

☒ A numerical value for number of cells or percentage (with statistics) is provided.

Methodology

Sample preparation

Flow cytometric nucleus sorting:
Nuclei of mouse spinal cords were isolated with the Nuclei Isolation Kit (Sigma-Aldrich, NUC101) according to the manufacturer's protocol with minor modifications. Briefly, mice were sacrificed with CO2 and perfused with cold PBS. Whole spinal cords were removed and stored at –80 °C. The tissue was mechanically dissociated with a scalpel on a petri dish placed on a cooled metal block. Tissue was added to 2 mL of EZ buffer (Sigma-Aldrich, NUC101) and further dissociated using a glass douncer (Sigma-Aldrich, D9063). After 5 minutes incubation on ice, the homogenate was centrifuged (500 × g, 5 minutes, 4 ° C) and the pellet was washed in 2 mL of EZ buffer, followed by two washing steps in nuclei incubation buffer (340 mM sucrose, 2 mM MgCl2, 25 mM KCl, 65 mM glycerophosphate, 5% glycerol, 1 mM EDTA, 1% bovine serum albumin). Nuclei were filtered using a 30 µm filter and directly stained with AF647-labeled NeuN antibody and 0.25 µg mL–1 propidium iodide (BioLegend, 421301). NeuN+ and NeuN– nuclei were sorted using a BD FACSAria III cell sorter (BD Biosciences) with a 70 µm nozzle.

Flow cytometric cell sorting:
For isolation of astrocytes and immune cells from spinal cord tissue, we incorporated the transcriptional inhibitor actinomycin D (ActD) throughout the workflow. Spinal cord tissue from EAE animals and healthy controls was collected in RPMI-1640 medium (PAN Biotech, P04-18500) supplemented with 25 mM HEPES (Gibco, 15630056) and 30 µM ActD (Cell Signaling, 15021S) after transcardial PBS perfusion. Tissue was dissociated into single-cell suspensions in 1 mg mL–1 collagenase A (Roche, 11088793001) and 200 IU mL–1 DNase I (Merck Millipore, 260913) using the gentleMACS Octo Dissociator (Miltenyi Biotec, program: Multi_F). The dissociated tissue was applied to a 70 µm cell strainer, and the filter was rinsed three times with RPMI-1640 supplemented with 25 mM HEPES and 3 µM ActD. Dissociated tissue was collected after centrifugation at 500 × g for 5 minutes, 4 °C and immune and glia cells were enriched using a discontinuous density gradient with Percoll PLUS (GE Healthcare, 17-5445-01). Isotonic Percoll solutions were prepared with HBSS and supplemented with 3 µM ActD. After centrifugation at 1350 × g, 4° C for 30 minutes, cells were collected from the interphase between the 30% Percoll and 70% Percoll layer. Cells were washed in FACS buffer (PBS, 1 mM EDTA, 1% BSA (Miltenyi Biotec, 130-091-376), 10

mM HEPES) at 650 × g, 4° C for 10 minutes. Nonspecific Fc receptor–mediated antibody binding was blocked by pre-incubation with TruStain FcX anti-mouse CD16/32 antibody (BioLegend, 101320) for 10 minutes at 4 °C before staining with surface antibodies in FACS buffer for 20 minutes at 4 °C. All antibodies used in this study are listed in Extended Data Table 7. Cells were washed and resuspended in FACS buffer supplemented with 0.4 U µL–1 RiboLock RNase Inhibitor (Thermo Fisher Scientific, EO0382) and 2.5 µM Helix NP Green (BioLegend, 425303) to exclude dead cells.

Immune cell isolation for flow cytometry:
Inguinal lymph nodes and spleen samples were homogenized through a 70 µm cell strainer and washed with PBS (500 × g, 5 minutes, 4 °C). Red blood cells were lysed as described above. Brain and spinal cord tissue were collected after transcardial PBS perfusion and dissociated into single cell suspensions in 1 mg mL–1 collagenase A and 0.1 mg mL–1 DNase I using the gentleMACS Octo Dissociator (program: Multi_F). The dissociated tissue was applied to a 70 µm cell strainer, and immune and glia cells were enriched using a discontinuous density gradient (GE Healthcare, GE17-0891-01). Cells were collected from the interphase as described above. Nonspecific Fc receptor–mediated antibody binding was blocked by pre-incubation with TruStain FcX anti-mouse CD16/32 antibody prior to staining of surface antibodies in Brilliant Stain Buffer (BD Biosciences) for 30 minutes at 4 °C. For staining of intranuclear proteins cells were fixed in 1X Fixation/Permeabilization working solution for 45 minutes at 4 °C, followed by incubation with antibodies targeting Nur77 or Ki67 in 1X Permeabilization buffer for 45 min at 4 °C (Invitrogen, 00-5523). All antibodies used in this study are listed in Extended Data Table 7. We excluded dead cells from the analysis by staining with Zombie Aqua, Green, Yellow and NIR Fixable Viability Stains (BioLegend, 423101, 423112, 423104, 423106) or 0.8 µM Alexa Fluor 750 NHS (Invitrogen, A20011). For the determination of absolute cell numbers, CD45high leukocytes and CD45med microglia were quantified using Precision Count Beads (BioLegend, 424902). Data were obtained using a BD Symphony A3 flow cytometer (BD Biosciences) and analyzed using FlowJo version 10.9 (BD Biosciences).

Instrument

For immune cell characterization by flow cytometry: BD Symphony A3 flow cytometer (BD Biosciences)
For flow cytometric nuclei/cell sorting: BD FACS Aria III Cell Sorter (BD Biosciences)

Software

For immune cell characterization by flow cytometry: FACSDiva software version 9.1 (BD Biosciences) and FlowJo version 10.9 (BD Biosciences).
For flow cytometric nuclei/cell sorting: BD FACSDiva v9.0.1 (BD Biosciences)

Cell population abundance

The frequencies of all analyzed cell/nuclei populations is depicted in the representative gating strategy.

Gating strategy

Gating strategies for all analyses are included in the Extended Data Figures.

☒ Tick this box to confirm that a figure exemplifying the gating strategy is provided in the Supplementary Information.
