# Supplementary material for: A GDF-15–GFRAL axis controls autoimmune T cell responses during neuroinflammation
Source: Nat Immunol. 2026 Jan 15;27(3):503–15. doi: 10.1038/s41590-025-02406-1 (PMC12956584; doi:10.1038/s41590-025-02406-1)

related to Figure 4c

Tyrosine hydroxylase (TH): GFP OE vs GDF-15 OE

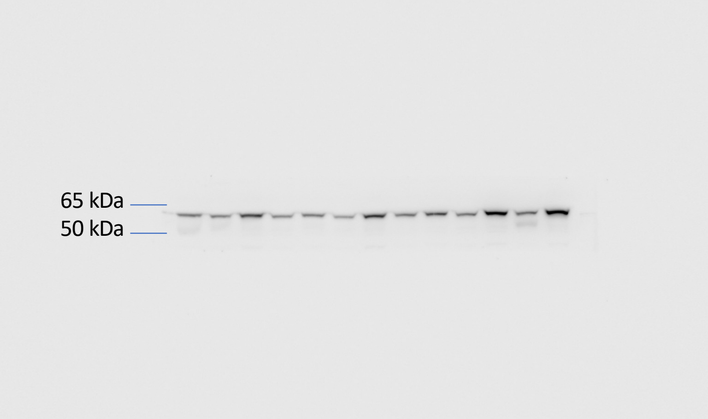

Vinculin (Vin): GFP OE vs GDF-15 OE

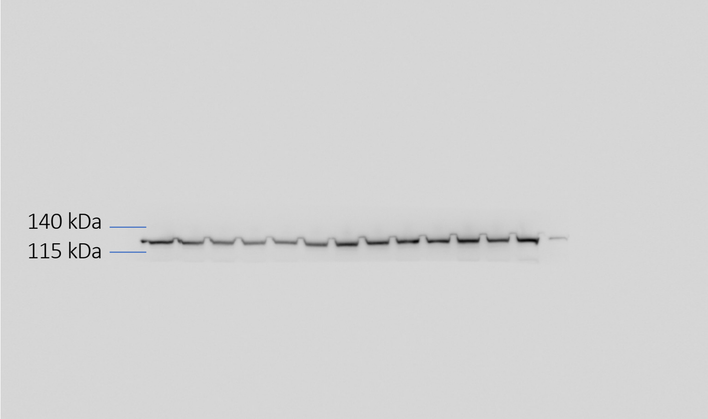

Tyrosine hydroxylase (TH): GFP OE, EAE vs GDF-15 OE, EAE

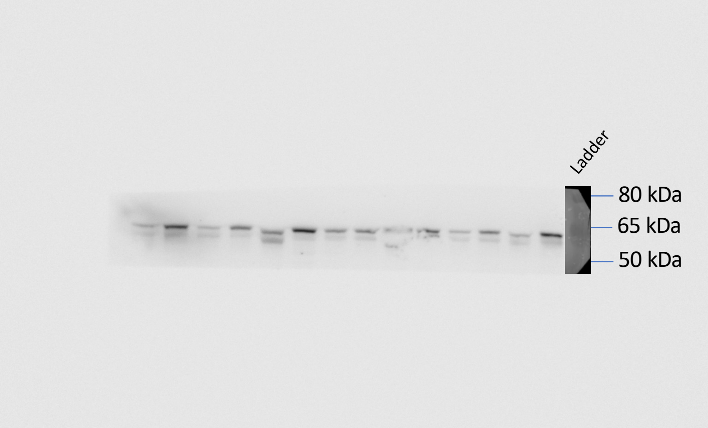

Vinculin (Vin): GFP OE, EAE vs GDF-15 OE, EAE

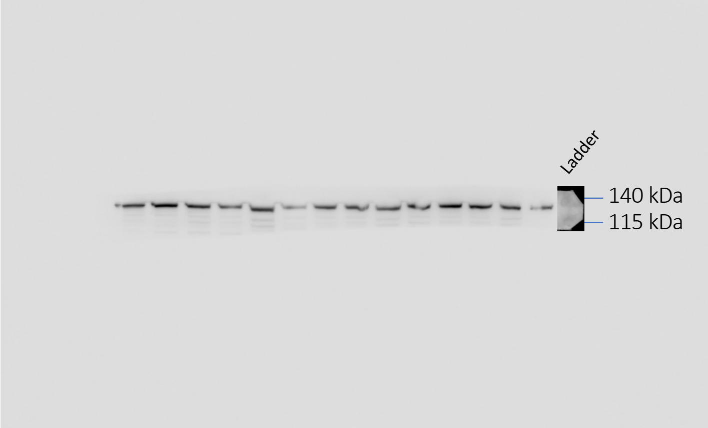

related to Figure 5k

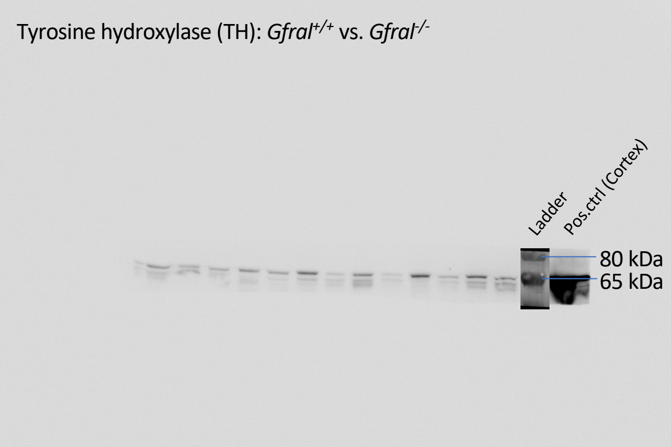

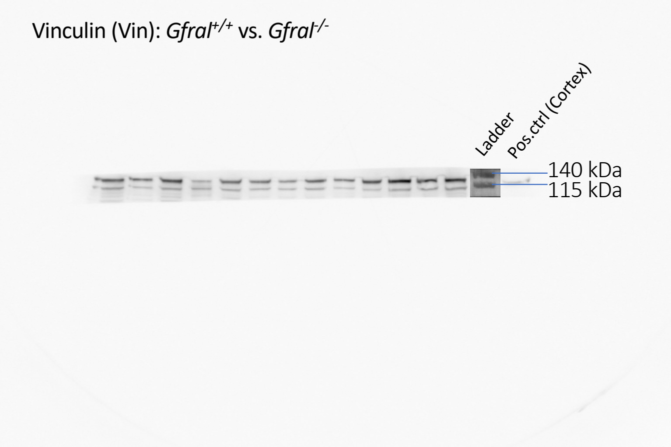

related to Figure 6h

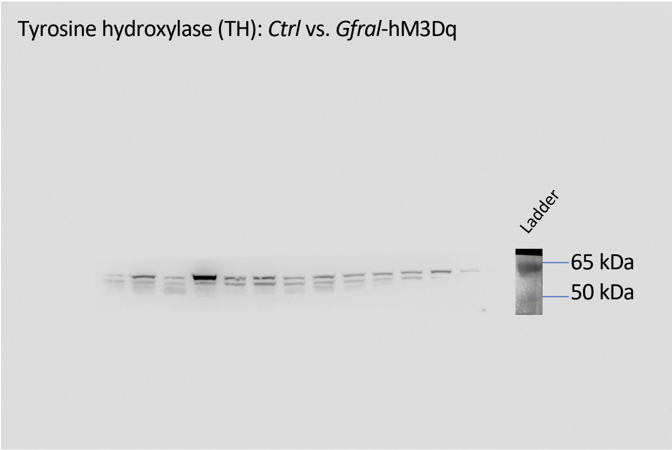

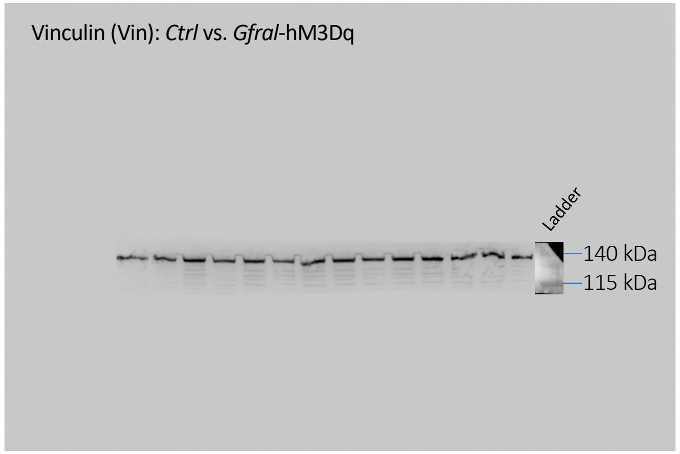

related to Extended Data Figure 6b

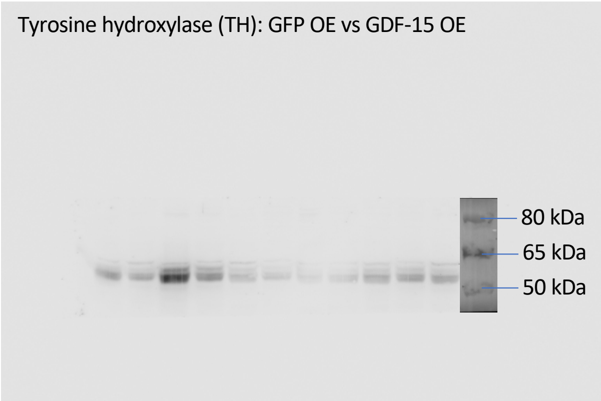

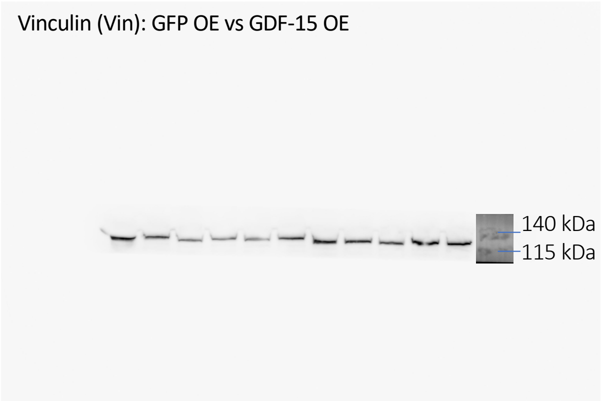

related to Extended Data Figure 10e

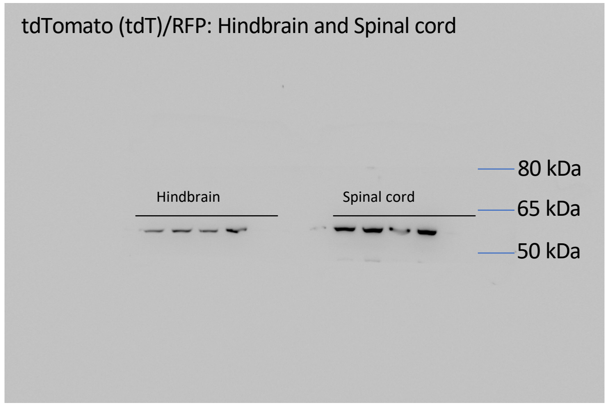

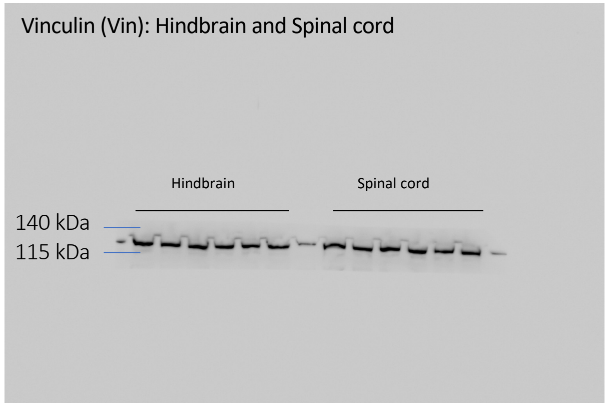

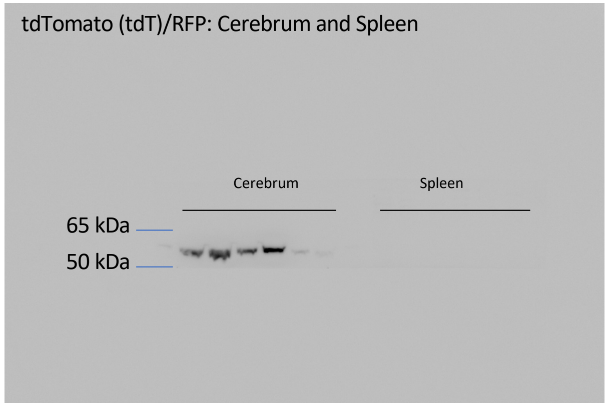

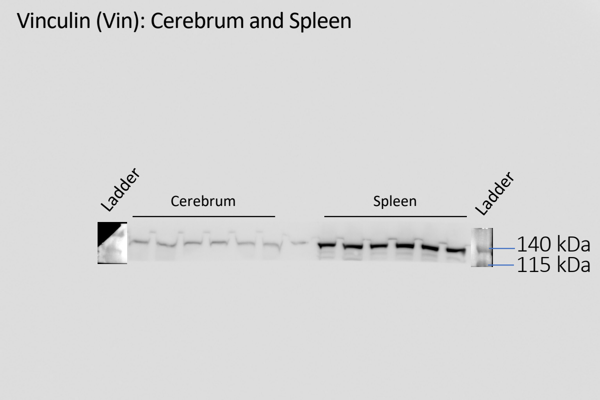

Representative RFP immunoblot with ladder

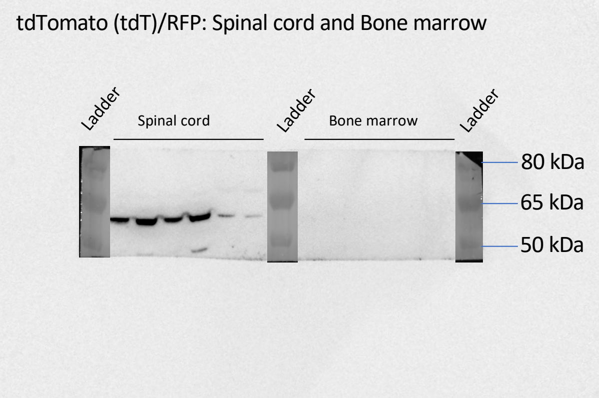

Supplement: Supplementary file 21 — Unprocessed immunoblot images. [file 41590_2025_2406_MOESM21_ESM.pdf]
